# Somatic rearrangements causing oncogenic ectodomain deletions of FGFR1 in squamous cell lung cancer

**DOI:** 10.1172/JCI170217

**Published:** 2023-11-01

**Authors:** Florian Malchers, Lucia Nogova, Martijn H.A. van Attekum, Lukas Maas, Johannes Brägelmann, Christoph Bartenhagen, Luc Girard, Graziella Bosco, Ilona Dahmen, Sebastian Michels, Clare E. Weeden, Andreas H. Scheel, Lydia Meder, Kristina Golfmann, Philipp Schuldt, Janna Siemanowski, Jan Rehker, Sabine Merkelbach-Bruse, Roopika Menon, Oliver Gautschi, Johannes M. Heuckmann, Elisabeth Brambilla, Marie-Liesse Asselin-Labat, Thorsten Persigehl, John D. Minna, Henning Walczak, Roland T. Ullrich, Matthias Fischer, Hans Christian Reinhardt, Jürgen Wolf, Reinhard Büttner, Martin Peifer, Julie George, Roman K. Thomas

**Affiliations:** 1 University of Cologne, Faculty of Medicine and University Hospital Cologne, Department of Translational Genomics, Cologne, Germany Germany.; 2 University of Cologne, Faculty of Medicine and University Hospital Cologne, Department I of Internal Medicine, Center for Integrated Oncology Aachen Bonn, Cologne Duesseldorf, Cologne, Germany.; 3 Mildred Scheel School of Oncology, Cologne, University Hospital Cologne, Medical Faculty, Cologne, Germany.; 4 University of Cologne, Faculty of Medicine and University Hospital Cologne, Institute of Pathology, Cologne, Germany.; 5 Department of Experimental Pediatric Oncology, University Children’s Hospital of Cologne, University Hospital Cologne, Medical Faculty, Cologne, Germany.; 6 University of Texas Southwestern Medical Center, Dallas, Texas, USA.; 7 Personalized Oncology Division, Walter and Eliza Hall Institute of Medical Research, Department of Medical Biology, The University of Melbourne, Parkville, Australia.; 8 DISCO Pharmaceuticals GmbH, Cologne, Germany.; 9 University of Berne and Cantonal Hospital of Lucerne, Cantonal Hospital of Lucerne, Lucerne, Switzerland.; 10 Département d’Anatomie et Cytologie Pathologiques, Grenoble, France.; 11 Institute for Diagnostic and Interventional Radiology, Faculty of Medicine and University Hospital Cologne, University of Cologne, Cologne, Germany.; 12 Institute of Biochemistry I, Medical Faculty, University of Cologne, Cologne, Germany.; 13 CECAD Cluster of Excellence, University of Cologne, Cologne, Germany.; 14 Centre for Cell Death, Cancer, and Inflammation (CCCI), UCL Cancer Institute, University College London, London, United Kingdom.; 15 Center for Molecular Medicine Cologne (CMMC), Cologne, Germany.; 16 Department of Hematology and Stem Cell Transplantation, University Hospital Essen, University Duisburg-Essen, Essen, Germany.; 17 Department of Head and Neck Surgery, Medical Faculty, University Hospital Cologne, Cologne, Germany.; 18 German Cancer Consortium (DKTK), partner site Heidelberg and German Cancer Research Center (DKFZ), Heidelberg, Germany.

**Keywords:** Genetics, Oncology, Drug therapy, Lung cancer

## Abstract

The discovery of frequent 8p11-p12 amplifications in squamous cell lung cancer (SQLC) has fueled hopes that FGFR1, located inside this amplicon, might be a therapeutic target. In a clinical trial, only 11% of patients with 8p11 amplification (detected by FISH) responded to FGFR kinase inhibitor treatment. To understand the mechanism of FGFR1 dependency, we performed deep genomic characterization of 52 SQLCs with 8p11-p12 amplification, including 10 tumors obtained from patients who had been treated with FGFR inhibitors. We discovered somatically altered variants of *FGFR1* with deletion of exons 1–8 that resulted from intragenic tail-to-tail rearrangements. These ectodomain-deficient FGFR1 variants (ΔEC-FGFR1) were expressed in the affected tumors and were tumorigenic in both in vitro and in vivo models of lung cancer. Mechanistically, breakage-fusion-bridges were the source of 8p11-p12 amplification, resulting from frequent head-to-head and tail-to-tail rearrangements. Generally, tail-to-tail rearrangements within or in close proximity upstream of *FGFR1* were associated with FGFR1 dependency. Thus, the genomic events shaping the architecture of the 8p11-p12 amplicon provide a mechanistic explanation for the emergence of FGFR1-driven SQLC. Specifically, we believe that FGFR1 ectodomain–deficient and *FGFR1*-centered amplifications caused by tail-to-tail rearrangements are a novel somatic genomic event that might be predictive of therapeutically relevant FGFR1 dependency.

## Introduction

Squamous cell lung cancer (SQLC) is the second most common lung cancer subtype and among the cancers with the worst prognosis (1). Unfortunately, genetically activated kinase targets that provide opportunities for effective treatment, such as those occurring in lung adenocarcinoma (e.g., *EGFR* mutations or *ALK* rearrangements), have so far not been identified in SQLC (2). The discovery of recurrent *FGFR1* amplifications (located on 8p11.23) had raised hopes that patients with such amplification might benefit from FGFR inhibition in this hard-to-treat cancer (3, 4). In a clinical trial testing this hypothesis by treating patients with 8p-amplified SQLC with the FGFR inhibitor BGJ398, approximately 11% of 8p11-p12–amplified tumors exhibited durable responses to single-agent FGFR inhibition (5). Thus, while the majority of these tumors are not dependent on FGFR, a minority require the catalytic activity of the kinase for their survival. The heterogeneous 8p11-8p12 amplification pattern might explain this observation to some extent, and a recent publication highlighted the role of *NSD3* as an oncogenic driver in SQLC (6, 7); however, a mechanistic reason for this phenotypic heterogeneity has so far been lacking. We therefore hypothesized that specific structural features of the 8p11 amplicon might explain FGFR1 dependency and performed an in-depth genomic and functional study of primary human squamous cell lung carcinomas from both patients treated with FGFR inhibitors as well as untreated patients, cancer cell lines, and patient-derived xenografts (PDXs).

## Results

### Tumors from patients with lung cancer who respond to FGFR inhibition exhibit tail-to-tail rearrangements within FGFR1.

**Figure 1:**
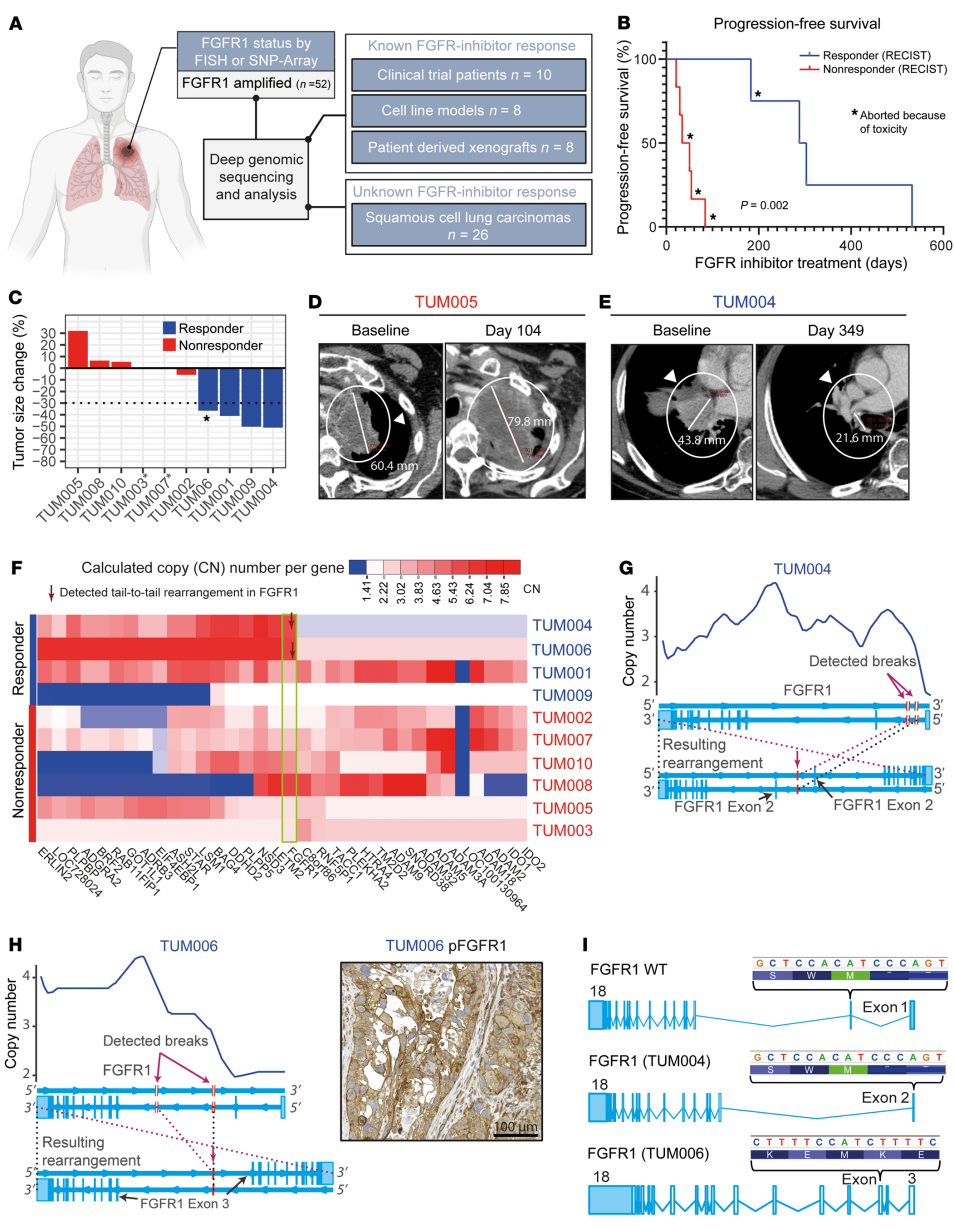
Tail-to-tail rearrangements in patients responding to FGFR inhibition. (**A**) Overview of the study cohorts. (**B**) Kaplan-Meier curve showing progression-free survival of patients with 8p11-amplified SQLC treated with the FGFR inhibitors BGJ398 or GW786034 (TUM006). *FGFR1* amplification was determined by FISH. Asterisk indicates that treatment was stopped because of toxicity. (**C**) Tumor volume change for patients with *FGFR1*-amplified SQLC treated with BGJ398 (Response Evaluation Criteria in Solid Tumors [RECIST] criteria). Tumor progression (red) and durable response (blue) following FGFR inhibition. TUM003 and TUM007 died during treatment with no sign of response. One patient (TUM006) was treated off-label (asterisk indicates that no RECIST data are available). Tumor shrinkage was estimated on CT scans (Supplemental Figure 1). (**D** and **E**) CT scans of patient TUM005 without a response and patient TUM004 with a durable response. (**F**) Copy number (CN) for 6 patients with progressive disease and 4 patients with a durable response to FGFR inhibition (5, 6). Red arrows indicate samples with tail-to-tail rearrangements within *FGFR1* (highlighted by a green frame). (**G**) Copy number plot magnified at the *FGFR1* locus (615x sequencing coverage). Patient TUM004 had a response to FGFR inhibition with BGJ398. Normal exon structure of *FGFR1* (middle), resulting in genomic rearrangement (bottom), and the location of the detected breaks are indicated by arrows, as are the resulting rearrangements. (**H**) Copy number plot magnified at the *FGFR1* locus (558x sequencing coverage). Tumor sample TUM006 was obtained from a patient responding to off-label treatment with GW786034. Normal exon structure (middle), the resulting genomic rearrangement (bottom), and the location of the detected breaks and resulting rearrangements are indicated by arrows. Image shows p-FGFR1 by IHC image (scale bar: 100 μm). (**I**) Transcript of *FGFR1* WT (ENST00000397091.9, top) and transcripts of *FGFR1* found in treatment-naive patient samples (middle and bottom) with possible ATG start codons (TAC motive from right to left; *FGFR1* is located on the negative strand). Light blue indicates UTRs.

We first collected 52 squamous cell lung carcinomas that were positive for *FGFR1* amplification, tested by FISH or Affymetrix SNP 6.0 arrays, and performed deep genomic sequencing (Figure 1A). We first analyzed treatment-naive biopsy specimens obtained from 10 patients with *FGFR1*-amplified SQLC (Figure 1, A and B). Nine of these patients had been treated with the FGFR inhibitor BGJ398 as part of a phase I clinical trial (NCT01004224), and 1 patient had been treated with pazopanib, a multi-kinase inhibitor that also inhibits FGFR (5, 6). Of these 10 patients, 4 had experienced a partial response (PR) (defined as tumor shrinkage of at least 30% of the maximal tumor diameter) lasting for 9 to 17 months (5), and 6 patients had progressive disease (PD) under treatment (Figure 1, A–E and Supplemental Figure 1; supplemental material available online with this article; https://doi.org/10.1172/JCI170217DS1). Given the small amount of available DNA obtained from these clinical specimens, we used a hybrid capture-based sequencing assay tailored to cover much of the genomic 8p11-8p12 locus, as well as 226 additional genes (6). We sequenced the 10 specimens with an average sequencing depth of 470×.

Although all specimens had been selected on the basis of the presence of *FGFR1* amplification determined by FISH as part of the inclusion criteria (Figure 1F and Supplemental Figures 2 and 3), we could not detect *FGFR1* amplification by this sequencing assay in all tumors (Figure 1F). In particular, we did not observe *FGFR1* amplification in the TUM009 specimen (compare Figure 1F and Supplemental Figure 3). Thus, *FGFR1* amplification was insufficient to predict the response to FGFR inhibition in this clinical cohort. However, to our surprise, in 2 specimens obtained from patients who had responded to FGFR inhibition, we found highly covered tail-to-tail rearrangements within *FGFR1*, which induced an abrupt copy number change within the ORF of *FGFR1* (TUM004 and TUM006 with 192 and 115 break-detecting reads, respectively) (Figure 1, G and H). In both cases, rearrangements were caused by 2 chromosomal breaks in close proximity, which led to an intrachromosomal fusion in a tail-to-tail manner and induced a loss of the first canonical *FGFR1* exons (Figure 1I and Supplemental Video 1). Upstream-located enhancer or silencer sequences and the complete promoter region of *FGFR1*, including the TATA box and transcription initiation region, were deleted, suggesting they might cause a functional deletion within FGFR1. In detail, the rearrangement in the sample TUM004 deleted upstream DNA sequences including exon 1 of *FGFR1*, which codes for the 5′-UTR. The canonical ATG start codon, located in exon 2, was unaffected and allowed the translation of an intact, full-length FGFR1 protein (Figure 1, G and I). The rearrangement in the sample TUM006 deleted upstream DNA sequences until exon 3 of *FGFR1* and therefore lacked the canonical ATG start codon of *FGFR1*, providing a noncanonical start codon in exon 5 (Figure 1, H and I). These specific/intrachromosomal tail-to-tail rearrangements were exclusively identified in patients who had responded to FGFR inhibition. Furthermore, in the TUM006 sample in particular, we observed robust staining for phosphorylated FGFR1 (p-FGFR1) by IHC analyses, indicating active transcription and catalytic activity of FGFR1 (Figure 1H, right). In this case, *FGFR1* could only be translated by using the first downstream in-frame ATG start codon located at exon 5, which would delete the immunoglobulin-like domain I and the acid box of FGFR1, which is known for its self-inhibitory function (8). On the contrary, tumors from patients with no clinical response to FGFR inhibition never showed such rearrangements and instead were found to have 3 different *PIK3CA* mutations (G118S, E545K, and H1047R; 3 of 6 patients). In summary, half of the patients with a response to FGFR inhibition had *FGFR1* amplification, which was caused by tail-to-tail rearrangements within the ORF of *FGFR1*, leading to retained protein expression and catalytic function of FGFR1.

### Intragenic somatic rearrangements causing ectodomain deletions of FGFR1 in primary SQLCs.

**Figure 2:**
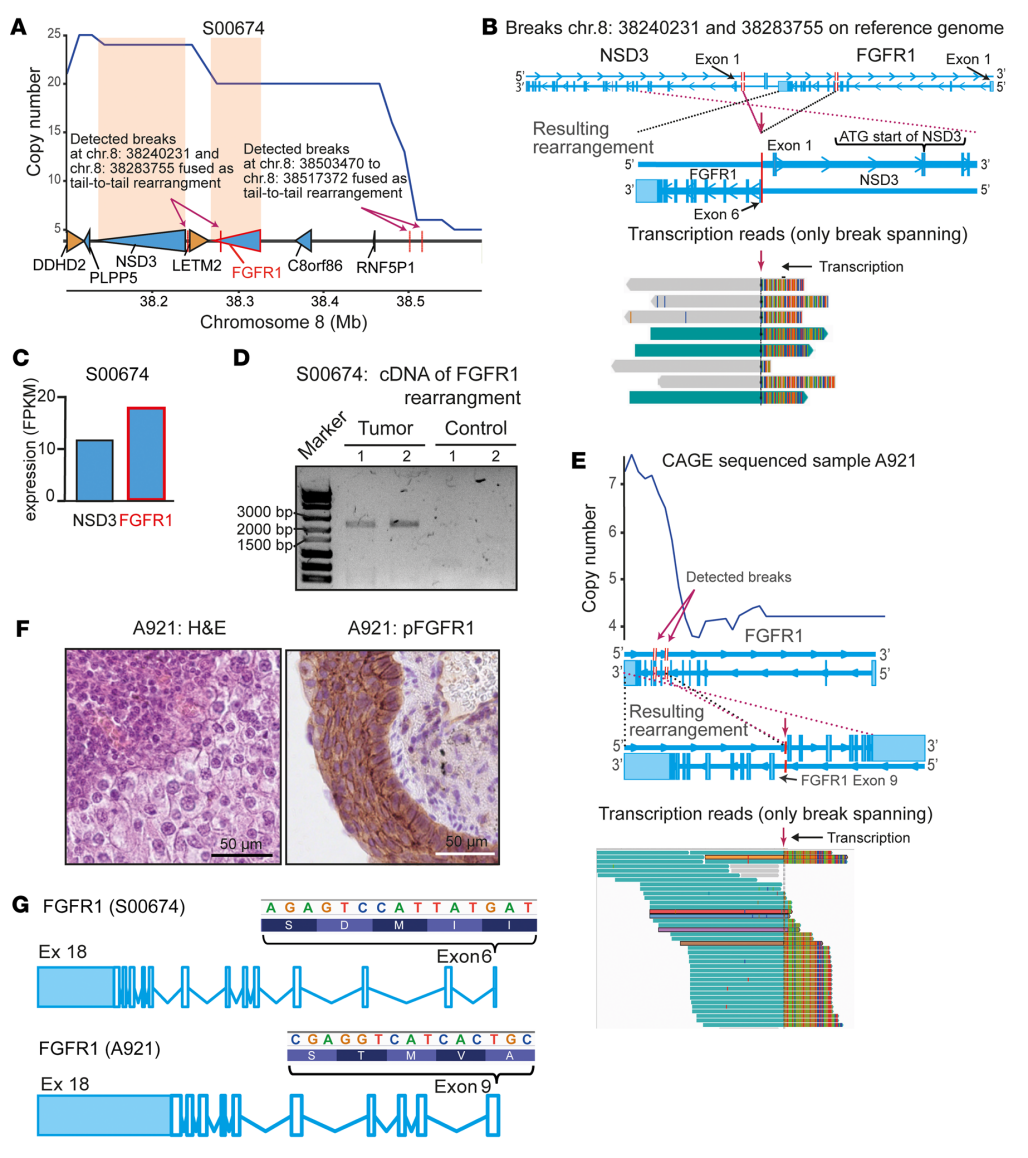
*FGFR1* tail-to-tail rearrangements in 8p11-8p12–amplified SQLCs. (**A**) Copy number plot (WGS, 30x coverage) of SQLC sample S00674 (*NSD3* and *FGFR1* are highlighted in orange). The reference genome and the location of genes (wedges) are indicated below (yellow, positive; blue, negative strand; detected breaks are indicated by arrows) (**B**) Normal exon structure of *NSD3* and *FGFR1* (top) is indicated. Resulting rearrangement (middle, magenta arrow indicates the tail-to-tail rearrangement; red bars indicate breaks and rearrangement) and breakpoint-spanning reads (from transcriptome sequencing) are shown (bottom). (**C**) Expression of NSD3-long and FGFR1α in sample S00674, as determined by transcriptome sequencing. (**D**) Electropherogram of a PCR using cDNA generated from tumor and normal (S00674) lung tissue. Two independent primer pairs covering the breakpoint were used (predicted band size: 1, 2.268 bp and 2, 2.407 bp). (**E**) Magnified copy number plot showing the genomic *FGFR1* locus (A921, 468× depth, unknown response to FGFR inhibition). Copy number (top), normal exon structure (middle), resulting genomic rearrangement (middle), and break-detecting transcriptomic sequencing reads (bottom, magenta arrow indicates the tail-to-tail rearrangement) are indicated. (**F**) Microscopic H&E-stained (left) and p-FGFR1 (right) images of the A921 sample. Scale bars: 50 μm. (**G**) Transcripts of *FGFR1* found in patient tumors with an unknown FGFR inhibitor response. Possible ATG start codons (TAC motive from right to left; *FGFR1* is located on the negative strand of the reference genome) and exons (Ex) (light blue areas are UTRs).

We next sought to validate our finding of ectodomain deletions in independent cohorts. Therefore, we performed an in-depth genomic characterization of 16 *FGFR1*-amplified cell lines and PDX models with a known in vitro and in vivo response to FGFR inhibition, and 26 additional *FGFR1*-amplified squamous cell lung carcinoma samples from patients with an unknown response to FGFR inhibition (Figure 1A). To our disappointment, we could not detect similar rearrangements in any of the cell lines or PDX models to validate our findings from patients that had been treated with FGFR inhibitors. However, in the cohort of *FGFR1*-amplified squamous cell lung carcinomas with an unknown response to FGFR inhibition, we detected similar rearrangements (S00674 and A921) in 2 patients. In the primary tumor S00674, we found a tail-to-tail rearrangement 150 kb upstream of the transcriptional start site of *FGFR1*. This rearrangement was again caused by 2 breaks in close proximity and led to an intrachromosomal fusion in a tail-to-tail manner (Figure 2A). A second tail-to-tail rearrangement within the ORF of *FGFR1* translocated closely to the transcriptional start site of *NSD3* (Figure 2, A and B), similar to the rearrangements observed in the 2 patients from the clinical trial who showed a response to FGFR inhibition (Figure 1, G–I). The tail-to-tail rearrangement upstream of *FGFR1* had a 3-fold higher coverage of reads across the breakpoint compared with the rearrangement within *FGFR1* (54 vs. 15 break-detecting reads), suggesting the second event had occurred later in the cancer genome evolution. The rearrangement in S00674 might be suggestive of inactivation of *FGFR1* and might also have particular relevance to *NSD3*. However, transcriptome sequencing revealed increased levels of *FGFR1* transcription compared with *NSD3*_long_ (Figure 2C). Furthermore, we found active transcription across the breakpoint affecting the *FGFR1* gene (Figure 2, B and D). In detail, the tail-to-tail rearrangement was an intrachromosomal event caused by 2 breaks within *FGFR1* (inside exon 6) and a noncoding region close to the transcriptional start site of *NSD3* (Figure 2B). The break led to deletion of *FGFR1* exons 1 to 5 and parts of exon 6, including the canonical ATG start codon and the genomic transcription initiation region. Although this finding was compatible with functional impairment of FGFR1, we confirmed transcription of the rearranged *FGFR1* gene by reverse transcriptase PCR (RT-PCR), using 2 different primer pairs (primer pair 1, 2.268 bp and primer pair 2, 2.407 bp; Supplemental Table 1), covering the breakpoint — partly exon 6, and exons 7 to 18 (including the FGFR1 kinase domain) — followed by sequencing (Figure 2D).

We speculated that the remaining ORF of *FGFR1* might be intact and that FGFR kinase activity could be preserved by use of an alternative start codon. This hypothesis was confirmed by an additional *FGFR1*-amplified adenosquamous carcinoma from another patient with an unknown response to FGFR inhibition. This patient also had a tail-to-tail rearrangement within *FGFR1* (sample A921, Figure 2E). The rearrangement was similar to the aforementioned rearrangements that we found in tumors from patients with a response to FGFR inhibition and led to deletion of exons 1 to 8 of *FGFR1* (Figure 2, E and G). Again, supporting our hypothesis, we found active transcription across the breakpoint (Figure 2E, bottom). In these tumor cells, we also observed positive staining for p-FGFR1 by IHC (Figure 2F), similar to what we had observed in the FGFR inhibitor–sensitive tumor (Figure 1H). However, using the next possible in-frame start codon for *FGFR1* translation would lead to deletion of the immunoglobulin-like domains I and II and the acid box in sample S00674 and the complete extracellular domain of FGFR1 in sample A921 (Figure 2G). In summary, we found 2 additional primary tumors with tail-to-tail rearrangements within *FGFR1*, suggesting that rearrangements within *FGFR1* are recurrent events in 8p11-p12–amplified lung cancer. Genome and transcriptome sequencing, validated by independent RT-PCR or IHC staining, enabled the discovery of rearrangements and expression of a N-terminally truncated version of FGFR1 ectodomain deficiency (up to exon 8). In these cases, the transmembrane and kinase domains of FGFR1 were not impaired, and the catalytic activity of FGFR1 was also preserved.

### Oncogenic potential of ectodomain-deficient FGFR1 variants.

**Figure 3:**
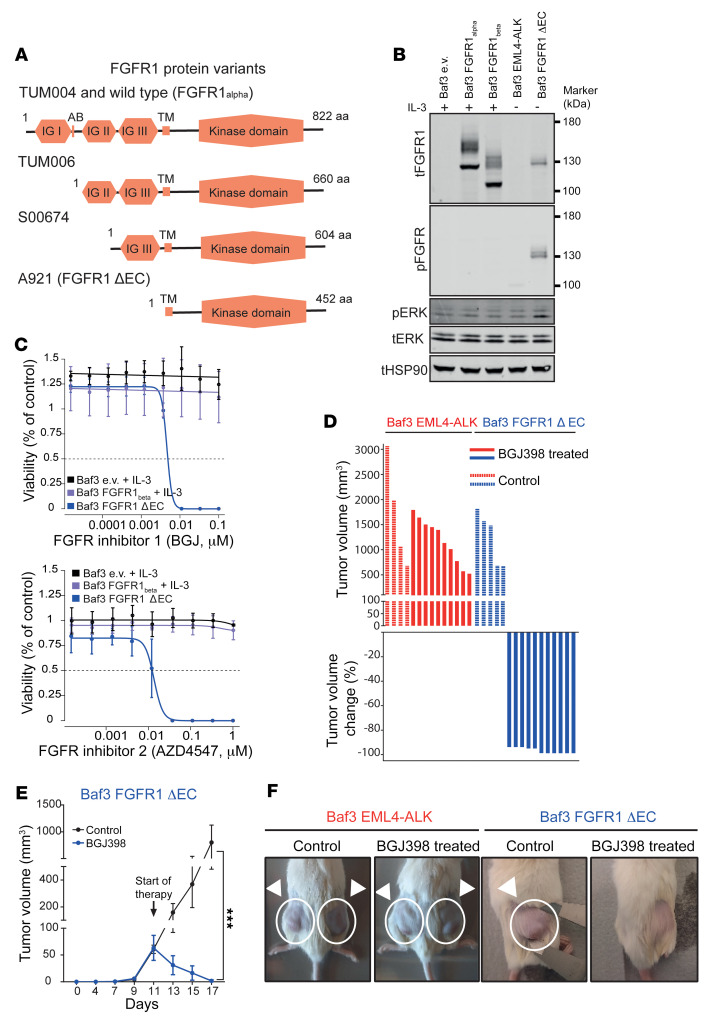
Oncogenic potential of an ectodomain-deficient version of FGFR1. (**A**) Overview of FGFR1 protein variants using the next possible in-frame ATG start codon of the transcripts shown in Figure 1I and Figure 2G. AB, acid box; TM, transmembrane domain. (**B**) Immunoblots of Ba/F3 cells transduced with retroviruses encoding ΔEC-FGFR1 and EML4-ALK (control), as well as parental Baf3 cells or cells transduced with empty vector (Baf3 e.v.), FGFR1α (Baf3 FGFR1_alpha_), FGFR1β, and (Baf3 FGFR1_beta_). Baf3 e.v, FGFR1α, and FGFR1β were cultured with IL-3. t, total. (**C**) Baf3 e.v., FGFR1β, and ectodomain lacking *FGFR1* (ΔEC-FGFR1, using an in-frame ATG in exon 9) were incubated with increasing concentrations of the FGFR inhibitor BGJ398 (BGJ, top) or the FGFR inhibitor AZD4547 (bottom) for 96 hours, with measurement of ATP content to determined viability. Baf3 e.v. and Baf3 FGFR1β cells were screened in the presence of IL-3, whereas Baf3 ΔEC-FGFR1 cells were screened without IL-3. (**D**) Quantification of xenograft tumor models engrafted with Ba/F3 cells expressing ΔEC-FGFR1 (blue) or EML4-ALK (red) following treatment with BGJ398 (20 mg/kg, q.d., red/blue bars) or vehicle (dashed red/blue bars). (**E**) Tumor volumes of a xenograft tumor model engrafted with Ba/F3 cells expressing ΔEC-FGFR1 that were treated with BGJ398 (20 mg/kg, q.d., blue curve) or vehicle (black curve), respectively, upon formation of palpable tumors. Tumor volumes were assessed as indicated and compared by 2-tailed *t* test. ***P < 0.0005. (**F**) Representative photographs of the xenograft models are shown before termination of the experiment.

The N-terminal part of the FGFR1 protein (comprising the ectodomain) is responsible for ligand specificity, as well as for receptor autoinhibition (8, 9). Furthermore, deletion of the whole FGFR1 ectodomain leads to ligand-independent dimerization (10). We therefore hypothesized that the transcribed ectodomain-lacking variants of FGFR1 might be oncogenic or cause oncogene dependency and thus sensitivity to FGFR inhibition. To test this hypothesis, we cloned *FGFR1* and deleted the ectodomain-encoding portion of the gene, up to all theoretically possible in-frame ATG start codons (termed ΔEC-FGFR1), as described in Figure 1, G–I, and Figure 2, B, E, and G (see also Figure 3A and Supplemental Figure 4). Ectopic expression of ΔEC-FGFR1 led to IL-3–independent cell growth and was sufficient to induce oncogenic transformation in murine Ba/F3 cells. Furthermore, it induced robust phosphorylation of ΔEC-FGFR1 (Figure 3B). By contrast, overexpression of 2 different WT FGFR1 versions (FGFR1α and FGFR1β) was not sufficient to induce IL-3 independence or FGFR1 phosphorylation (Figure 3B and Supplemental Figure 5). In these experiments, we noted that the bands of ΔEC-FGFR1 migrated at a larger-than-expected protein size (Supplemental Figure 5). However, through the use of 1 HA-Tag version of ΔEC-FGFR1, mass spectrometry studies, and mRNA transcript analysis, we were able to formally exclude the possibility of endogenous murine FGFR transactivation in these murine cells (Supplemental Figures 5 and 6). Thus, while we can’t fully explain the size difference in immunoblotting, our analyses indicate constitutive FGFR1 kinase activation by amino-terminal deletion of FGFR1. Furthermore, ΔEC-FGFR1 caused FGFR1 oncogene dependency in Ba/F3 cells, as evidenced by their high sensitivity to FGFR inhibition by the FGFR inhibitors BGJ398 and AZD4547 (Figure 3C). By contrast, bromodomain inhibition, which would inhibit the oncogenic driver NSD3 (7), had no differential viability effects on Ba/F3 cells expressing ΔEC-FGFR1 compared with control Baf3 cell lines (Supplemental Figure 7). Furthermore, Baf3 cells, transformed by ΔEC-FGFR1, formed tumors in a xenograft tumor model. These tumors were highly sensitive to FGFR inhibition using the FGFR inhibitor BGJ398 in vivo (Figure 3D). On the contrary, FGFR inhibition had no effect on Baf3 cells transformed by the oncogene EML4-ALK, thus confirming the selectivity of the compound (Figure 3, E and F). Consequently, somatic genomic amino-terminal deletion of all extracellular domains like IG I and acid box (which have a self-inhibitory function) and IG II and IG III (which are ligand-binding and ligand-specify domains) caused a novel oncogenic variant of FGFR1. This oncogenic variant included the transmembrane and kinase domain of FGFR1, ΔEC-FGFR1, and caused therapeutically tractable FGFR dependency in vitro and in vivo.

### Distinct rearrangements within the 8p11-p12 locus associate with sensitivity to FGFR inhibition in cancer models lacking ectodomain-deficient FGFR1.

**Figure 4:**
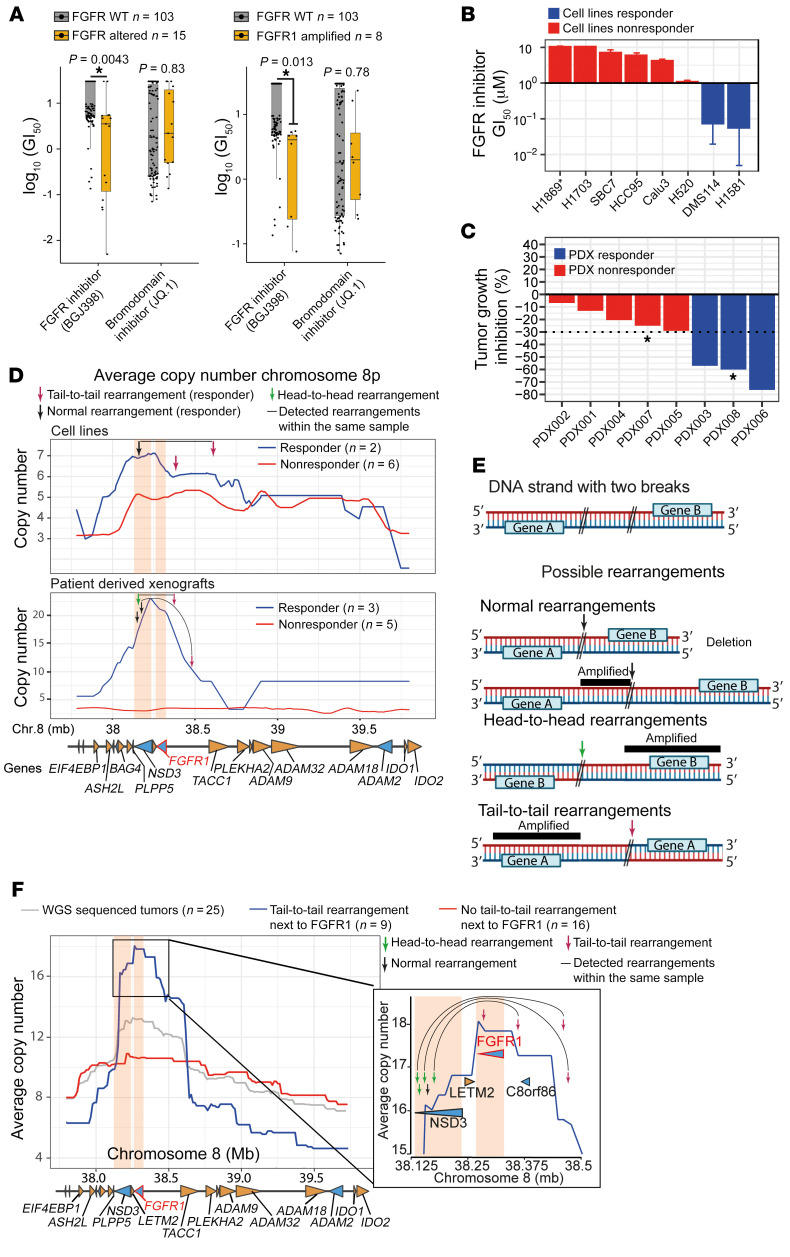
Rearrangements associated with FGFR inhibitor sensitivity. (**A**) GI_50_ of 118 cancer cell lines treated with BGJ398 or JQ.1, sorted according to the presence or absence of somatic *FGFR* gene family alterations (left) or the presence or absence of *FGFR1* amplification (excluding other FGFR alterations, right). *P < 0.05. (**B**) Average GI_50_ values of 8 cell lines (>1 μM in red; <1 μM in blue) treated with the FGFR inhibitors BGJ398 or AZD4547. (**C**) Average tumor growth reduction of 8 PDX tumor models treated with 20 mg/kg BGJ398 or vehicle control (resistant red, tumor reduction <30 %; sensitive blue, tumor reduction >30 %). Asterisks indicate samples provided by Weeden et al. (13). (**D**) Average copy number of 6 cell lines resistant to (red) and 2 cell lines sensitive to (blue) FGFR inhibition (top panel) and 5 resistant (red) and 3 sensitive (blue) PDX tumor models (bottom panel). *NSD3* and *FGFR1* are highlighted (orange). Locations of genes (wedges) are indicated below (yellow, positive strand; blue, negative strand). Rearrangements in samples from responders are indicated. (**E**) Illustration of 3 possible rearrangements and their impact on copy number (see Supplemental Video 1 for a detailed explanation). (**F**) Average copy number of 25 8p-amplified primary SQLC specimens with unknown responsiveness to FGFR inhibition (whole-genome sequenced). Data are plotted together (gray, *n* = 25) or with (400 kb range, blue, *n* = 9) or without observed tail-to-tail rearrangements (>1 mb range, red, *n* = 16) before *FGFR1*. Magnification of the amplification peak is shown (right, blue group, *n* = 9). Only rearrangements observed within the ORF of *NSD3* or *FGFR1* are indicated (arrows). Corresponding rearrangements within the same sample are also indicated, if located within the same sample (black lines) and if detected within the magnified area (arrows: head-to-head in green, normal in black, and tail-to-tail in red).

Using an orthogonal approach, we next sought to investigate the role of genomic rearrangements and their underlying impact on genome structure and copy number and their potential impact on FGFR inhibition. Therefore, we performed a small-molecule inhibitor screen of 118 cancer cell lines against FGFR- and bromodomain inhibitors (Figure 4A). Confirming previous studies (3, 11), we found that cell lines bearing genomic *FGFR* alterations (including FGFR mutations and amplifications) were frequently sensitive to FGFR inhibition (*P* = 0.005). Furthermore, while 8p amplification was predictive of sensitivity to FGFR inhibition (*P* = 0.02), it failed to predict sensitivity to bromodomain inhibition (*P* = 0.8), which was recently described to associate with 8p amplification affecting *NSD3* (Figure 4A) (7). Of note, some 8p-amplified cell lines were particularly sensitive to FGFR inhibition, while others were not (Supplemental Figure 8), suggesting a predominant dependency on the kinase activity in these cases. We focused our analysis on 8 *FGFR1*-amplified lung cancer cell lines treated with the small-molecule inhibitors BGJ398 and AZD4547 (Supplemental Figure 9). Of these 8 cell lines, 6 were resistant (half-maximal growth-inhibitory concentration [GI_50_] >1 μM)) and 2 were sensitive (GI_50_ <1 μM) to FGFR inhibition. As mentioned above, we did not find tail-to-tail rearrangements within the *FGFR1* gene itself or genomic evidence of ectodomain-deficient versions of the FGFR1 kinase that we had observed in the patients with a response to FGFR inhibition, as well as in the cohort of untreated squamous cell lung carcinomas (see above).

In order to identify additional genomic mechanisms driving FGFR1-dependent cancers, we therefore plotted and analyzed the average copy number for these 2 groups (Figure 4D and Supplemental Figures 10 and 11). Similar to the copy number profiles generated from primary, patient-derived tumors (Supplemental Figure 2), we observed centered amplification on *FGFR1* and *NSD3* in the sensitive cell lines, whereas resistant cell lines showed no clear center of the amplicon (Figure 4D and Supplemental Figures 9–11). Despite the lack of intragenic *FGFR1* rearrangements, we identified tail-to-tail rearrangements close to *FGFR1* in both FGFR inhibitor–sensitive cell lines, which drove the observed amplification pattern (Figure 4D). In detail, we found tail-to-tail rearrangements close to *FGFR1* in H1581 cells (chr8: 38.595.657 bp) and in DMS114 cells (chr8: 38.382.689 bp), but not in any of the resistant cell lines (Figure 4D). Furthermore, we detected a deleterious *NSD3* break in the FGFR inhibitor–sensitive cell line H1581. This rearrangement fused exon 15 of *NSD3* to exon 10 of *ANK1*, creating an out-of-frame fusion, which strongly suggests inactivation of *NSD3*. Thus, the H1581 cell line exhibited 2 rearrangements, which were also found in patient TUM009, who had responded to FGFR inhibition (Figure 1F, Figure 4D, and Supplemental Figure 2). Together, the findings suggest that tail-to-tail rearrangements upstream of *FGFR1* lead to the observable of *FGFR1*/*NSD3*-centered amplification patterns. This goes along with sensitivity to FGFR inhibition and co-occurring deletion of the oncogenic NSD3 SET domain (Figure 4D) (7). These findings provide further support for a key role of FGFR1 in driving sensitivity to FGFR inhibition. In summary, our study shows that (in addition to ectodomain-deficient versions of FGFR1, resulting from intragenic rearrangements) tail-to-tail rearrangements close to *FGFR1* may drive FGFR1-centered amplification, favoring *NSD3* deletion and FGFR1 dependency.

We next collected 85 PDX models and tested them for 8p11-8p12 amplification by FISH (12). We identified 8 models harboring 8p amplifications and treated them in vivo with the FGFR inhibitor BGJ398 (Figure 4C and Supplemental Figure 12). Of these PDX models, 5 were resistant and 3 were sensitive to FGFR inhibition (Figure 4C) (13). We sequenced all 8 PDX models and searched for intragenic deletions of *FGFR1* that might cause ectodomain-deficient *FGFR1*. We also plotted the average chromosomal gene copy number of the sensitive and resistant groups in this cohort (Figure 4D). Similar to the findings in the cell lines described above, we did not observe intragenic *FGFR1* rearrangements or other signs of ectodomain-deficient *FGFR1*. However, similar to the pattern of amplification observed in patient samples and cancer cell lines, FGFR inhibitor–sensitive PDX specimens had *FGFR1*/*NSD3*-centered amplification, whereas FGFR-deficient samples showed no clear center of amplification (Figure 4D and Supplemental Figure 11). The amplification pattern observed in FGFR inhibitor–sensitive PDX samples was again driven by tail-to-tail rearrangements close to *FGFR1* and rearrangements within *NSD3* (Figure 4, D and E). In detail, we found 2 tail-to-tail rearrangements close to *FGFR1* (PDX003, chr8: 38.371.080 bp; PDX008, chr8: 38.481.135 bp) and disrupting *NSD3* rearrangements in all 3 sensitive PDX models (PDX003, deletion of *NSD3* exons 16 to 24; PDX006, deletion of *NSD3* exons 9 to 24; PDX008, deletion of *NSD3* exons 9 to 24) (Figure 4D). In particular, the detected rearrangements in PDX003 and PDX008 showed strong similarities to those found in the FGFR inhibitor–responsive tumor from patient TUM009 and the cancer cell line H1581, indicating recurrent rearrangements associated with FGFR1 dependency (Figure 4D and Supplemental Figures 2, 10, and 11). By contrast, in resistant PDX models, 8p amplifications were neither centered on *FGFR1* nor focal within the 8p11-8p12 locus (Figure 4D). Furthermore, we found a destructive head-to-head rearrangement in the insensitive/resistant PDX001 model, deleting the whole *NSD3* gene and exons 6 to 18 of *FGFR1* and thus the kinase domain of FGFR1. This suggests that neither *FGFR1* nor *NSD3* was the target of 8p amplification (Figure 4E and Supplemental Figure 11).

In a pooled analysis of cell lines, PDX models, and patient-derived specimens, tail-to-tail rearrangements in or close to *FGFR1* occurred in 7 of 9 (78%) “responders” and in 3 of 12 (25%) “nonresponders” (for 5 cell lines, only whole-exome sequencing data were available) and were associated with sensitivity to FGFR inhibition (*P* = 0.03, Fisher’s exact test). Furthermore, we found destructive *NSD3* rearrangements in 5 of 9 FGFR inhibitor–sensitive samples (56%, deleting exons 6 to 23; *P* = 0.007, Fisher’s exact test), but not even in 1 of 12 insensitive/resistant models or patients. Thus, in 8p-amplified tumors, tail-to-tail rearrangements affecting *FGFR1* were associated with sensitivity to FGFR inhibition, while destructive rearrangements within *NSD3* make a functional role of NSD3 in these tumors unlikely. Combined with the tumors with amino-terminal truncation of *FGFR1*, the carcinomas with tail-to-tail rearrangements within FGFR1 or in close proximity to the FGFR1 gene locus may thus constitute the overall population of FGFR1-dependent SQLC with sensitivity to FGFR inhibition.

### Tail-to-tail rearrangements close to the FGFR1 gene in primary human lung cancer.

We wondered whether these distinct amplification patterns could also be observed in primary 8p11-p12–amplified lung tumors with an unknown response to FGFR inhibition. We therefore performed an in-depth reevaluation of the genome-sequencing data of the 26 aforementioned 8p11-p12–amplified primary tumors, in which we had also discovered the amino-terminally truncated versions of FGFR1. For 25 of these 26 samples, we were able to calculate the chromosomal gene copy number (14–16). As expected, the average copy number of all 25 samples revealed high-amplitude amplification that was centered on *FGFR1* and the adjacent *NSD3* gene (FDR *q* = 1.3 × 10^–38^) (gray line in Figure 4F and Supplemental Figure 13). We next screened for tail-to-tail rearrangements occurring close to the transcription start site of *FGFR1*. We therefore chose a 400 kilobase (kb) region upstream of *FGFR1*, based on the cell lines and PDX specimens that were sensitive to FGFR inhibition. Confirming our findings in cell lines and PDX models, we found that 9 of 25 samples harbored a tail-to-tail rearrangement within this region (36%) and 1 with co-occurring intragenic rearrangements or an amino-terminal deletion of *FGFR1* (Figure 2A). Remarkably, only these 9 samples with tail-to-tail rearrangements drove the observed *FGFR1*/*NSD3*-centered amplification pattern (blue line in Figure 4F, left panel, and Supplemental Figure 13). These specimens exhibited *FGFR1*-centered and focal amplification (550 kb), whereas the amplified region was 3 times larger (1.7 Mb) in tumors without distinct tail-to-tail breaks (red line in Figure 4F, left panel). Of note, in 5 of these 9 specimens with tail-to-tail rearrangements close to *FGFR1*, we observed an additional rearrangement within the *NSD3* gene. These breaks were mainly head-to-head breaks (4 of 5 cases) and led to the deletion of exons encoding the SET domain of *NSD3* (required for oncogenic transformation) and thus to an amplification pattern favoring *FGFR1* exclusively (7) (Figure 4F, right panel, and Supplemental Figure 14). These tumors also exhibited increased expression of the short isoform of *NSD3* lacking the catalytic SET domain (Supplemental Figure 15). Thus, in 9 of 25 specimens of 8p-amplified lung cancer, we found tail-to-tail rearrangements close to the transcriptional start site of *FGFR1*. This rearrangement, in particular, induced a copy number gain of *FGFR1*. Furthermore, 5 of these tumors had an additional rearrangement within *NSD3* that caused focal amplification, centered exclusively on *FGFR1*, similar to the observed amplification pattern in FGFR inhibitor–sensitive cell lines, PDX models, and patient-derived specimens (Figure 4, D–F, and Supplemental Figure 2). In summary, tail-to-tail rearrangements upstream of *FGFR1* and presumably destructive rearrangements in *NSD3* can be frequently observed in lung cancer cell lines, PDXs, and primary squamous cell lung carcinomas. They can be clearly separated from other 8p11-p12–amplified lung tumors and are nearly identical to rearrangements observed in patients with a response to FGFR inhibition. These distinct rearrangements exclusively favor the *FGFR1* gene and can be associated with FGFR inhibitor sensitivity.

### A mechanistic explanation for the emergence of 8p amplification and FGFR1 dependency.

**Figure 5:**
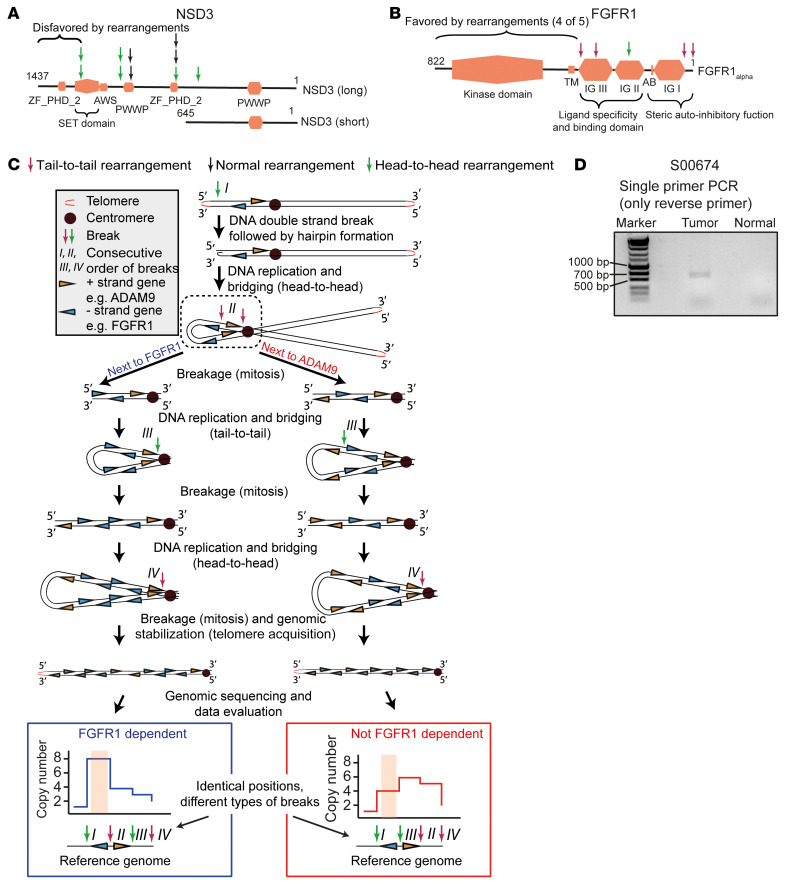
*FGFR1* dependency and consecutive order of genomic rearrangements. (**A**) Domain architecture of NSD3-long (top, 1 to 1,437 aa) and NSD3-short (bottom, 1 to 645 aa) and all detected rearrangements within all study groups (*n* = 52; arrow color indicates the type of rearrangement). (**B**) Domain architecture of FGFR1α (1 to 822 aa, *n* = 52; arrow color indicates the type of rearrangement. (**C**) Schematic overview of 2 BFB mechanisms forming 8p11-p12 amplifications, differing only in the consecutive order of genomic rearrangements. (**D**) Electropherogram of a PCR across a head-to-head rearrangement (S00674, tumor and matched normal DNA) using only 1 primer.

As described above, we identified 10 genomic rearrangements within *NSD3* and 5 within *FGFR1* (Figure 5, A and B). While the observed breaks in *NSD3* were disrupting (mainly deleting) the oncogenic SET domain, breaks in *FGFR1* were mainly tail-to-tail rearrangements (4 of 5), amplifying the oncogenic kinase domain (Figure 5, A and B) (10, 17). We furthermore found that in all 25 whole-genome–sequenced and *FGFR1*-amplified tumors, telomeric losses were accompanied by frequent intrachromosomal head-to-head and tail-to-tail rearrangements (Figure 4, D and F, Figure 5, A–C, and Supplemental Figures 16 and 17). Each of these rearrangements arose from 2 breakpoints, which were mainly located on the same chromosome and in close proximity to each other. The palindromic nature of a head-to-head rearrangement, induced by 2 breaks (S00674, chr8: 36.417.298 bp and chr8: 36.418.018 bp) was validated by use of a single-primer PCR (794 bp) (Figure 5D). Thus, the characteristic features of telomeric losses, clipped read orientation, and copy number alterations with at least 8 copy number segments indicated a breakage-fusion-bridge (BFB) amplification mechanism (Figure 5C) (18, 19). By applying these criteria, we reliably identified BFBs as the underlying cause of 8p amplification in 44% (11 of 25) of the primary 8p11-p12–amplified tumors. In 56% of the samples, a BFB mechanism was uncertain (48%) or excluded (8%) (19). Following the assembly logic of BFB as the underlying mechanism, we were able to correctly reconstruct the 8p amplicon of 1 tumor, considering only the observed genomic breaks (Supplemental Figures 17 and 18). However, although the BFB mechanism explains the emergence of 8p amplification in SQLC, it is unlikely to have predictive value for *FGFR1* dependency by itself (Figure 5C). It suggests that 8p11-p12 amplification emerges over several cell generations, ending in a dominant clone or a heterogeneic tumor cell population. In summary, 8p amplifications in SQLC are caused by BFBs in a large fraction of cases. In a subset of 8p11-p12–amplified tumors, intrachromosomal tail-to-tail rearrangements close to the *FGFR1* transcription start site are associated with rearrangements in *NSD3* and cause *FGFR1*-centered amplification with frequent functional inactivation of *NSD3*.

## Discussion

Here, we report 2 types of genomic alterations that associate with FGFR1 dependency and thus sensitivity to FGFR inhibition: intragenic rearrangements of *FGFR1* leading to ectodomain-deficient variants of FGFR1 on the one hand, and tail-to-tail rearrangements close to the *FGFR1* gene that cause *FGFR1-*centered amplification (and, in several instances, genomic inactivation of *NSD3*) on the other.

Intragenic *FGFR1* rearrangements causing ectodomain-deficient *FGFR1* were detected in 4 *FGFR1*-amplified lung cancer samples (8% of the *FGFR1*-amplified samples used in this study). The tail-to-tail rearrangements occurred within the *FGFR1* ORF and deleted various portions of the gene. To our surprise, these versions of *FGFR1* were still transcribed by making use of a noncanonical in-frame ATG start codon. Furthermore, we discovered that these ectodomain-deficient/-lacking versions of FGFR1 were oncogenic in vitro and in vivo and led to sensitivity to FGFR inhibition. The mechanism of ligand-independent dimerization by FGFR1 variants lacking the ectodomain has been reported previously and may explain the phenotype observed by us (10). However, somatic genomic alterations that cause such variants have not to our knowledge been described to date. Of note, *FGFR2* alterations causing deletion of the extracellular domain were reported recently in cholangiocarcinoma (20).

Furthermore, tail-to-tail rearrangements close to *FGFR1* frequently cause *FGFR1-*centered amplification in tumors lacking intragenic *FGFR1* rearrangements and the encoded ectodomain-deficient versions of the kinase. These rearrangements are similarly associated with sensitivity to FGFR inhibition and frequently accompanied by destructive rearrangements of *NSD3*, thus arguing in favor of a functional relevance of *FGFR1*, rather than of *NSD3*, in driving the oncogenic state in the affected tumors. We were able to show that this pattern of amplification was always caused by similar distinct rearrangements, and thus we could link these rearrangements to FGFR inhibitor sensitivity.

Finally, we found that 8p amplifications in SQLC are frequently caused by BSBs (18, 19). This mechanism of amplification induces head-to-head, followed by tail-to-tail, intrachromosomal rearrangements within the 8p arm. The consecutive order of rearrangements underlies an evolutionary process of tumor development. It also explains the frequently described and observed heterogenous pattern of amplification of 8p11-p12 and may also explain the generally limited degree of response to FGFR inhibition as well as early tumor progression under therapy.

Together, our findings suggest that intragenic tail-to-tail rearrangements in *FGFR1*, causing ectodomain-deficient versions of the kinase as well as tail-to-tail rearrangements close to *FGFR1* that drive *FGFR1-*centered amplification, may define SQLCs with therapeutically relevant FGFR dependency.

## Methods

### Human lung tumor specimens.

We collected a total of 26 fresh-frozen SQLC tumor samples, which were provided by multiple collaborating institutions as fresh-frozen tissue specimens, frozen sections, or genomic DNA extracted from fresh-frozen material (21). All tumor samples were pathologically assessed to have a purity of at least 60% and no extensive signs of necrosis. Additionally, these tumor samples were reviewed by at least 2 independent expert pathologists, and the diagnosis of SQLC was histomorphologically confirmed by H&E staining and IHC (21). Matching normal material was provided in the form of EDTA-anticoagulated blood or adjacent nontumorigenic lung tissue. The matched normal tissue was confirmed to be free of tumor contaminants by pathological assessment. Furthermore, tumor and matching normal material were confirmed to be acquired from the same patient by SNP 6.0 array and sequencing analyses. Patient material was stored at –80°C.

### Genome and transcriptome sequencing.

Next-generation sequencing data on whole exomes or whole genomes were analyzed using our in-house–developed pipeline, which has been used and described in previous large-scale cancer genome sequencing projects (16, 22–24). Briefly, the data were processed by aligning sequencing reads to a reference genome (NCBI build 37/hg19) using bwa-mem (0.7.13-r1126; https://github.com/lh3/bwa), masking potential PCR duplicates and regions of overlapping read pairs, and collecting various count statistics, which are used to call mutations and, for whole genomes, genomic rearrangements. Finally, Sclust (24) was used for sample purity estimation and copy number analysis. RNA-Seq data were analyzed using TRUP (25).

### ARCHER sequencing.

RNA was extracted from formalin-fixed, paraffin-embedded (FFPE) material using the Maxwell RSC in combination with the Maxwell RSC RNA FFPE Kit (Promega) according to the manufacturer’s instructions. Removal of genomic DNA was performed with the TURBO DNA-Free Kit (Thermo Fisher Scientific), and both transfer RNA (tRNA) and RNA were quantified using the Qubit RNA HS Assay Kit (Thermo Fisher Scientific). Analysis of RNA integrity was done with the 4200 TapeStation System (Agilent Technologies).

For fusion detection, the Archer FusionPlex Lung Panel (ArcherDX) was used according to the manufacturer’s instructions, with 200 ng tRNA input for library preparation. Purified libraries were quantified using the KAPA Library Quantification Kit (Roche). Sequencing was performed on the NextSeq System (Illumina), and results were analyzed using Archer Suite Analysis, version 6.2.7 (ArcherDX). Since the Archer Suite Analysis software is not able to label inversions, bam files were extracted and loaded directly into the Integrative Genomics Viewer (IGV) to visualize tail-to-tail breaks.

### Computational analysis.

Copy numbers were visualized with GISTIC (26), web-based interactive builder from the MD Anderson Cancer Center and ROBOCOP. To summarize the copy number information in the FGFR1 region across samples, a view window of 2MB around the FGFR1 locus was split into 10,000 bins. The available chromosome segment–based copy number data were then mapped onto these bins for each of the samples. Next, the per-bin means of the copy numbers for all samples within a certain condition were calculated, and the rolling mean with a window size of 10 (for whole-genome sequencing data) and 500 of these numbers was determined to reduce noise in the cap analysis of gene expression (CAGE) data. The process of determining ROlling Binmeans Of COPy numbers is defined here as the ROBOCOP algorithm. Inference of the BFB mechanism was performed using algorithms and criteria from Zakov et al. (19). Given a chromosome segmentation and sequence of segment copy number values n at the (amplified) FGFR1 locus, the approach estimates an approximate copy number sequence n’ by applying palindromic BFB transformations to an initial wild-type segmentation. A similarity/distance between the sequences is measured as the Poisson likelihood of observing sequence n given the approximation n’. Using the provided Java application, BFB inference was done in 2 steps: (a) estimating n’ (java bfb.BFB_Algo counts:n mode:substring model:Poisson maxError:0) and (b) converting n’ into a sequence of BFB transformations (java bfb.BFB_Algo counts:n’ mode:search). Following Zakov et al. (19), only amplicons with a BFB approximation of at least 8 segments were considered to be high confidence (*n* = 11), whereas amplicons with fewer than 8 segments and/or no support by fold-back inversions were too ambiguous to reliably infer BFB as the underlying mechanism (*n* = 14).

### Cell culture and reagents.

Cell lines were obtained from the American Type Culture Collection (ATCC), DSMZ (German Collection of Microorganisms and Cell Cultures), and in-house and were cultured using either RPMI or DMEM high-glucose media, supplemented with 10%–20% FCS. Adherent cells were routinely passaged by washing with PBS buffer followed by incubation in trypsin/EDTA. Trypsin was inactivated by addition of culture medium, and cells were plated or diluted accordingly. Suspension cell lines were passaged by suitable dilution of the cell suspension. All cells were cultured at 37°C in 5% CO_2_. *FGFR1* amplification was determined by SNP 6.0 array, and/or whole-genome sequencing, and/or whole exome sequencing, and/or CAGE sequencing or downloaded from COSMIC (https://cancer.sanger.ac.uk/cosmic).

Compounds were obtained from Selleck Chemicals, Tocris Bioscience, or MilliporeSigma. They were diluted in DMSO, aliquoted, and stored as 10 mM stocks at –80°C.

### Viability assays.

An initial cell line screen was performed on 384-well plates using 500–2,000 cells (depending on the cell line). For validation, cell lines were plated as triplicates in sterile 96-well plates at a density of 1,500 (adherent cells) and 5,000 (suspension cells) cells/well as described previously (6). After 24 hours of incubation, compounds were added at increasing dosages, ranging from 30 to 0.00003 μM, together with a separate DMSO control. After 96 hours, relative cell viability was determined by comparing the ATP content of each well, as assessed by CellTiter Glo Assay (Promega), with the content of the DMSO control. Finally, GI_50_ were calculated by R programming.

### PDX models.

In total, we performed *FGFR1* FISH on 35 PDX models from EpoBerlin, 39 PDX models from Crown Bioscience, and 3 samples from Moro Massimo as previously described (12). In addition, we established 8 PDX models by subcutaneously implanting 2–3 mm^3^ tumor pieces from biopsies (transported in DMEM media) together with 5–10 μL Matrigel (Corning) into NSG mice. We identified 8 *FGFR1*-amplified models and performed CAGE sequencing (6). CAGE-sequenced samples with confirmed *FGFR1* amplification were treated with BGJ398 (Tocris Bioscience, 20 mg/kg, dissolved in 33% PEG 300, 5% glucose, with fresh stock prepared every week and stored at 4°C) or vehicle (33% PEG 300, 5% glucose) and administered orally to mice daily, with at least 3 animals per group.

### Xenograft model.

IL-3–independent Baf3 cells transformed with ΔEC-FGFR1 or EML4-Alk were subcutaneously injected into both flanks of NSG mice. Mice with palpable tumors were randomly grouped and treated daily with either BGJ398 (Tocris Bioscience, 20 mg/kg, dissolved in 33% PEG 300, 5% glucose, with fresh stock prepared every week and stored at 4°C) or vehicle (33% PEG 300, 5% glucose).

### Patient samples.

From the clinical trial reported by Nogova et al., we were able to receive 8 formalin-fixed biopsies, taken before treatment (NCT01004224) (5). Three of these patients had a PR and 5 showed stable or progressive disease. Given the small amount of tissue, we were limited to CAGE sequencing (6). In addition, the University of Berne and Cantonal Hospital of Lucerne provided 1 sample from a patient who had a clear response following off-label treatment with pazopanib. All samples were confirmed to be 8p-amplified by *FGFR1* FISH and CAGE sequencing.

### Break validation by PCR.

To verify expression over the breakpoint and to validate the tail-to-tail rearrangement, as well as the ΔEC-FGFR1 variant, we generated cDNA from 1 μg total RNA from sample S00674 and its corresponding normal sample. cDNA was generated using the SuperScripIII kit (Invitrogen, Thermo Fisher Scientific) following manufacturer’s instructions, followed by PCR using the break-spanning primers 191_F1_S00674/203_R1_amplify_FGFR1 (Figure 2D) and 193_F2_S00674/ 204_R2_amplify_FGFR1 (Figure 2D and Supplemental Table 1).

A head-to-head rearrangement was validated in this sample by a nested PCR using only 1 primer per PCR run (given the palindromic nature of this break). In the first run, we used the primer 261_6_R1. In the second run (4 μL template from first PCR), we used the primer 262_6_R2 (Supplemental Table 1). The expected band size was 794 bp (Figure 5D, based on the whole-genome sequencing data).

### ΔEC-FGFR1 cloning.

cDNA of H1581 cells (100 ng) was used to amplify *FGFR1* by attB-overhang primers and flip it into pDONR.221 using the BP-clonase (Invitrogen, Thermo Fisher Scientific). Bacterial transformation of the competent *E*. *coli* strain DH5α (Invitrogen, Thermo Fisher Scientific) was carried out according to the manufacturer’s instructions. Single clones were sequenced from mini-preparation of plasmid DNA using the NucleoSpin Mini Kit (Machery Nagel). For midi-preparation of plasmid DNA we used the NucleoBond Xtra Midi EF Kit (Machery Nagel). The different *FGFR1* variants were generated by side using Gibson Assembly and the primers 234_F_ΔEC-21-FGFR1, 235_F_ΔEC-30-FGFR1, 236_F_ΔEC-85-FGFR1, 237_F_ΔEC-144-FGFR1, and 211_R_FGFR1_GA (Supplemental Table 1).

### Virus production.

Virus was produced as described previously (6).

### Immunoblotting.

Cells were washed with cold PBS and lysed in RIPA Lysis Buffer supplemented with protease (Roche) and phosphatase (Calbiochem) inhibitor cocktails. After a 20-minute incubation on ice, lysates were centrifuged at 18,000*g* for 25 minutes. The protein concentration in supernatants was measured using a bicinchoninic acid (BCA) Protein Assay (Thermo Fisher Scientific). Equivalent amounts of protein (30–60 μg) were denatured and separated on 4%–12% SDS-PAGE gels after blotting on nitrocellulose membranes (Amersham Hybond-C Extra). The following antibodies were used for immunoblotting: β-actin (MP Bioscience); HSP90 (CS4877S, Cell Signaling Technology); p-FGFR (Tyr653, Tyr654, CS3676, Cell Signaling Technology); HA-TAG (CS3724, Cell Signaling Technology); p-AKT (Ser473, CS4370, Cell Signaling Technology); AKT (CS2920, Cell Signaling Technology); p-ERK (CS4370, Cell Signaling Technology); total ERK (CS4696, Cell Signaling Technology); total FGFR1 (ab76464, Abcam); and conjugated antibodies against rabbit and mouse (MilliporeSigma).

### IHC analysis.

Tissues were fixed in 4% PBS-buffered formalin and embedded in paraffin (FFPE). IHC was performed as described previously on 3 μm slides with specific antibodies against p-FGFR1 (Abnova, Y154).

### Statistics.

For tail-to-tail rearrangement statistical significance, a 2-tailed Fisher’s exact test was used. For tumor growth inhibition and inhibitor screens statistical significance, a 2-tailed *t* test was used. A *P* value of less than 0.05 was considered significant.

### Study approval.

The study was reviewed and approved by the IRB of the Department I of Internal Medicine, Center for Integrated Oncology Cologne/Bonn, University Hospital Cologne and Ethics Committee of the University of Cologne (reference no. 06-037, 09-172, 10-275, and 13-091; biomarker research 10-242). The animal experiments were reviewed and approved by the local animal ethics committee of North Rhine-Westphalia in Düsseldorf (Landesamt für Natur, Umwelt und Verbraucherschutz Nordrhein-Westfalen, LANUV NRW). The protocols were performed according to the recommendations of the Federation of European Laboratory Animal Science Association (FELASA).

### Data availability.

Whole-genome and transcriptome sequencing data on human specimens have been deposited in the European Genome-phenome Archive (accession code EGAS00001005059). Values for all data points in graphs can be found in the Supplemental Supporting Data Values file (patient data file names are: TUM001 = A1106, TUM002 = A1107, TUM003 = A1109, TUM004 = A1116, TUM005 = A1114, TUM006 = p68, TUM007 = A1115, TUM008 = A1782, TUM009 = A1113, TUM010 = A1111. Cell line file names are according to cell line names. PDX file names are: PDX001 = A2328_LU1155, PDX002 = A2336_LU1775, PDX003 = A2322_14573, PDX004 = A2323_14574, PDX005 = A2337_LU2504, PDX006 = A3010, PDX007 = S02753, PDX008 =S02754. File names of samples with unknown FGFR inhibitor response are: A921, S00062, S00141, S00148, S00186, S00204, S00321, S00338, S00408, S00422, S00454, S00473, S00504, S00509, S00674, S00996, S01112, S01143, S01189, S01225, S01233, S01251, S01327, S01472, S01661, S01743). Additional data are available from the corresponding author upon request.

## Author contributions

FM and RKT conceptualized the study. MHAVA, L Meder, CB, RM, and JMH designed the study methodology. FM and LN performed experiments. MHAVA, L Maas, JB, CB, and JG conducted visualization studies. AHS, CEW, ID, JR, JS, KG, MP, PS, SMB, and TP performed experiments. RTU, MLAL, JW, HW, and RKT acquired funding. LG, GB, and SM were responsible for project administration. FM and RKT supervised the study. FM and RKT wrote the original draft of the manuscript. JG, OG, EB, MLAL, JDM, MF, HCR, JW, RB, and RKT reviewed and edited the manuscript.

## Supplementary Material

Supplemental data

Supplemental video 1

Supporting data values

## Version 1 08/22/2023

In-Press Preview

## Version 2 11/01/2023

Electronic publication
