# Supplementary material for: Somatic rearrangements causing oncogenic ectodomain deletions of FGFR1 in squamous cell lung cancer
Source: J Clin Invest. 2023 Nov 1;133(21):e170217. doi: 10.1172/JCI170217 (PMC10617767; doi:10.1172/JCI170217)
Supplement: Supplemental data [file jci-133-170217-s043.pdf]

Supplementary Figure Legends

Figure S1 *Tumor response upon FGFR inhibition*

Chest computer tomography (CT) scan of patients treated with BGJ398 as part of a phase-I clinical trial. TUM006 was a 79-year-old patient with smoking history. The patient revealed a FGFR1-amplified squamous cell lung cancer with a detected FGFR break, leading to extracellular FGFR1 deletion. The patient refused chemotherapy but agreed to Pazopanib off-label use (800mg). CT scans have been carried out before treatment start (left picture, baseline) and after 8 weeks of treatment.

Figure S2 *Average copy number of clinical trial patients*

Average copy number of 5 patients with progressive disease (red) or 4 patients with durable response (blue) to FGFR inhibition (Nogova, Malchers et al.). NSD3 and FGFR1 are highlighted in orange. The reference genome and the location of genes (wedges) are indicated below (yellow, positive; blue, negative strand). Arrows indicate detected head-to-head and tail-to-tail rearrangements with potential clinical relevance.

Figure S3 *Dual-color FISH of TUM009*

Dual-color FISH (FGFR1, green; Chr. 8 (control), red) of TUM009 before FGFR inhibitor treatment (left). Picture was taken with a 600-fold magnification. Evaluated FISH data of sample TUM009 (right).

Figure S4 *Cloning of delta FGFR1*

Exons coding for extracellular domains of *FGFR1* were deleted according to natural occurring in-frame ATG start codons (see also Primers, table 1) and based on observed rearrangements. Four different *FGFR1* variants were cloned using Gibson Assembly approach into pBabe puro backbone (P68, S00674, Virtual, and FGFR1ΔEC, images from https://prosite.expasy.org/, top right, bottom right). *FGFR1* open reading frames

were digested and sanger sequenced. Different *FGFR1* variants in pBabe were double digested with HindIII and NheI and ran on 1% agarose gel (left).

Figure S5 *Oncogenic delta FGFR1 expression causes phosphorylated protein band shift*

Immunoblot of wild type BAf3 cells (Baf3 wt) or Baf3 cells transduced with empty vector (e.V.), FGFR1 wild type alpha and beta (FGFR1 alpha, FGFR1 beta), and Baf3 cells transduced in 4 complete independent experiments with delta FGFR1 (delta EC-FGFR1(1), delta EC-FGFR1(2), delta EC-FGFR1(3), and HA-Tagged delta EC-FGFR1 (HA)). Stained for pFGFR (Tyr653, Tyr654), HA, and Actin. Baf3 e.V., wt, FGFR1 alpha, and beta demonstrated no transformed phenotype and were cultured with IL3 (+). Baf3 delta EC-FGFR1(1), delta EC-FGFR1(2), delta EC-FGFR1(3), and delta EC-FGFR1(HA) demonstrated a transformed phenotype (highly FGFR inhibitor sensitive) and were cultured without IL3 (-). Marker indicates different molecular weights in kilo Dalton (kDa).

Figure S6 *Electropherogram*

Electropherogram of a RT-PCR with mouse/human specific *FGFR1*-Primers binding extra- (left) and intracellular (right) located domains of FGFR1

Figure S7 *Screening of Baf3 cells against Bromodomain inhibitors*

Baf3 e.v., FGFR1beta and ectodomain lacking FGFR1 (FGFR1 ΔEC, using an in-frame ATG in exon 9, NM_015850) were incubated with increasing concentrations of the Bromodomain inhibitors AZD5153 (left) or JQ1 (right) for 96 hours, measuring ATP content to determine viability. Baf3 e.v. and Baf3 FGFR1beta cells were screened in the presence of IL-3, whereas Baf3 FGFR1 ΔEC was screened without IL-3.

Figure S8 *FGFR inhibitor sensitive cell lines*

FGFR1 amplified cells were rescreened against two FGFR inhibitors (BGJ398 and AZD4547) in a 96-well format measuring the ATP levels after 96 hours. GI50 were plotted accordantly (left). Results were validated by crystal blue staining because of the H520 cell line with GI50 values GI~1µM (right).

Figure S9 *FGFR inhibitor sensitivity correlates with 8p11-p12 amplification pattern*

H1581, SBC7, DMS114, HCC95, H520, H1703 and Calu3 cells were plated on 96-well plates. One the next day the cells were treated with increasing concentrations of the FGFR inhibitors BGJ398 and AZD4547 and after 96 hours cell viability was measured by cell titer glow. Each curve represents three different experiments that each consists of three replicates (left graph). The facility score was calculated for each cell line by dividing the amplitude by the length of the 8p11-p12 amplicon (amplitude and length were taken from 6.0 array data, the cell line with the highest score (H1581) was normalized to 1; x-axis, right graph) and plotted against BGJ398 sensitivity (y-axis, right graph). The sensitivity was calculated from Annexin V data (each cell line was plated on 6 cm dish and treated 72h with 100nM BGJ 398. Afterwards the supernatant was collected. Attached cells were trypsinized and pooled with the supernatant, followed by FACS sorting) by normalizing the most sensitive cell line (H1581) to 1, and from cell viability assays (see above) by normalizing the most sensitive cell line (H1581) to 1 (highest screened concentration (10µM) minus IC50 value). The average of both experiments was plotted on the y-axis.

Figure S10 *Individual copy number plot per gen and cell line*

Calculated copy number from genomic sequencing data were plotted per gene and cell line (for the 8p11-p12 locus) individually. The highest copy number within the open reading frame of the gene was plotted. A green frame highlights *FGFR1*. Heat map was

generated using the web-based interactive builder from the MD Anderson Cancer Center.

Figure S11 *Individual copy number plot per gene and PDX*

Calculated copy number from genomic sequencing data were plotted per gene and PDX (for the 8p11-p12 locus) individually. The highest copy number within the open reading frame of the gene was plotted. A green frame highlights *FGFR1*. Green arrow indicates a detected head-to-head rearrangement within *FGFR1* (leading to a c-terminal deletion of FGFR1 and e.g. NSD3). Heat map was generated using the web-based interactive builder from the MD Anderson Cancer Center.

Figure S12 *Inhibitor response of 8p-amplified patient derived xenografts*

8p-amplified patient-derived xenograft tumor models treated in vivo with 20 mg/kg BGJ398 or with a vehicle control. Tumor volumes were assessed each day. Statistical significance was assessed by two-tailed t-test.

Figure S13 *Copy number analysis of 25 8p11-p12 amplified SQLC samples*

Genomic Identification of Significant Targets in Cancer (GISTIC) analysis of 25 8p11-p12 amplified squamous cell line carcinomas. Copy number data were extracted from tumor vs. matched normal whole genome sequencing data.

Figure S14 *Amplification pattern of samples with a head-to-head break in NSD3*

Four samples with a head-to-head break in *NSD3* and a tail-to-tail break close to the transcription start site of *FGFR1* are shown. Red square on the chromosome 8 indicate the plotted area (top). Locations of genes are indicated with green (forward strand) or yellow (reverse strand) boxes together with chromosomal location (bottom, hg19, plotted with ROBOCOP.

Figure S15 *Transcription of NSD3 long vs. short in samples with/without NSD3 break*

Expressional analysis of 21 squamous cell lung carcinomas with (n=4, orange) or without (n=17, grey) destructive breaks in *NSD3*. FPKM values were extracted from whole transcriptome sequencing data, normalized and plotted.

Figure S16 *8p11-12 amplified lung cancer carcinomas show frequent telomeric losses*

Copy number analysis of 35 lung carcinomas (CLCGP, Seidel et al.) based on SNP 6.0 array data. Red square on the chromosome 8 indicates the illustrated area (top). Location of *FGFR1* is indicated with black bars (hg18, illustrated with integrative genome viewer). From this dataset we sequenced 25 squamous cell lung cancer carcinomas which all demonstrated telomeric loss.

Figure S17 *Copy number plot and detected breaks of an FGFR1 amplified sample*

Purity corrected copy number data extracted from whole genome sequencing data (S00148), plotted against genomic location (bottom) together with all detected genomic breaks on the 8p arm. Head-to-head rearrangements (dark red bars), tail-to-tail breaks (light red bars) and one normal rearrangement (blue bars) are indicated.

Figure S18 *Precise reconstruction of the 8p-locus taking only detected breaks into account*

The precise order of rearrangements (based on coverage) and the BFB evolution of the 8p-arm are indicated by letters starting from the centromere for the sample S00148 (left). Copy number plots of whole-genome sequencing data obtained from a tumor/normal pair of one patient (60x sequencing depth). Estimated copy number (calculated, top), integral copy number (purity corrected, middle), and observed breaks and copy number plotted according to the BFB mechanism (bottom) for this tumor specimen (S00148)

Table S1 *List of used primers*

Suppl. Figure 1:
Tumor response upon FGFR inhibition

TUM005

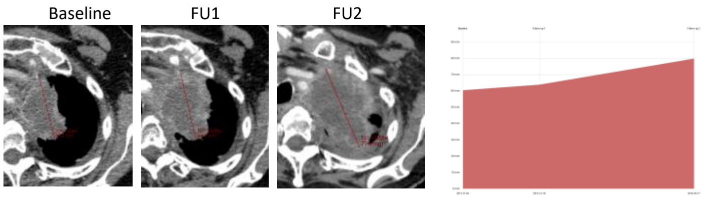

TUM010

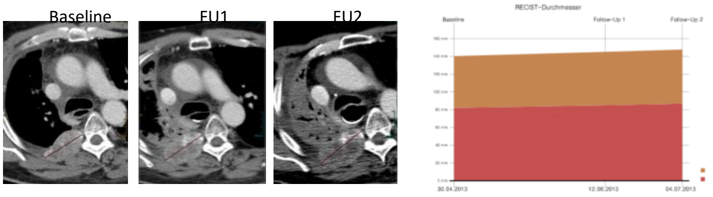

TUM006

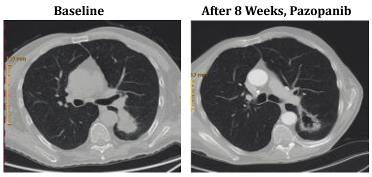

TUM001

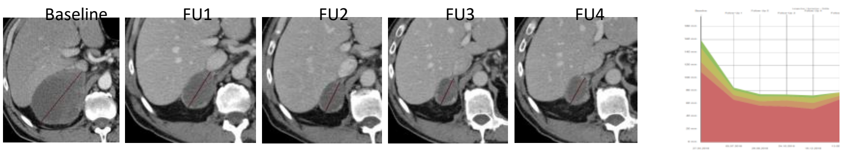

TUM009

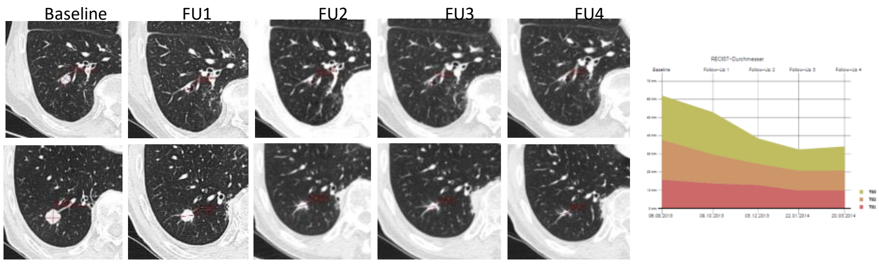

TUM004

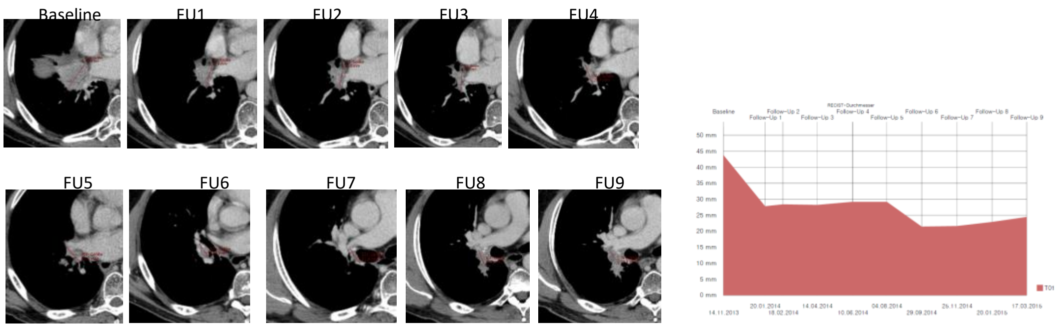

Suppl. Figure 2:
Average copy number of clinical trial patients

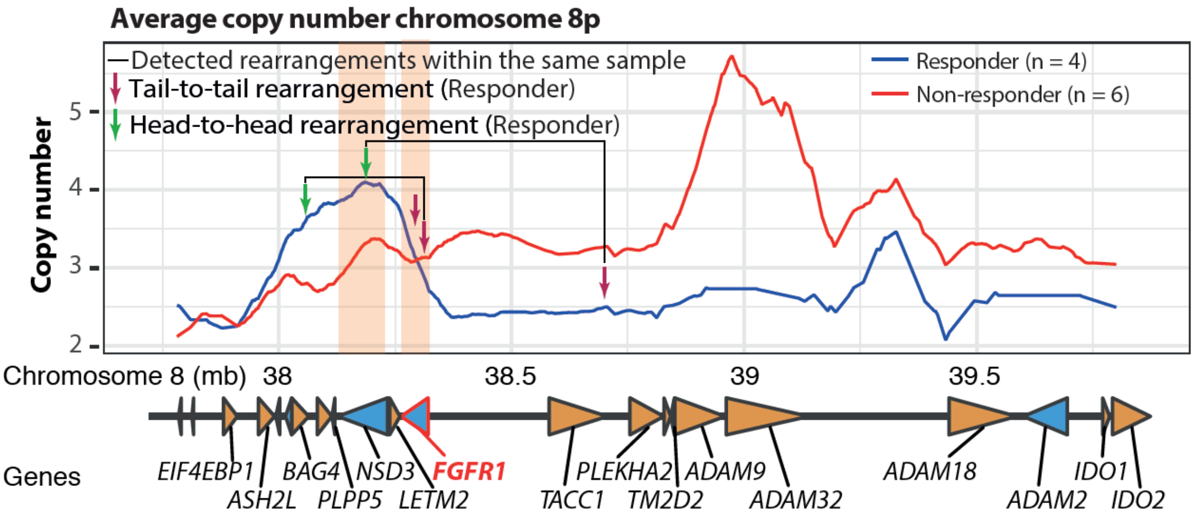

Suppl. Figure 3:
Dual-color FISH of TUM009

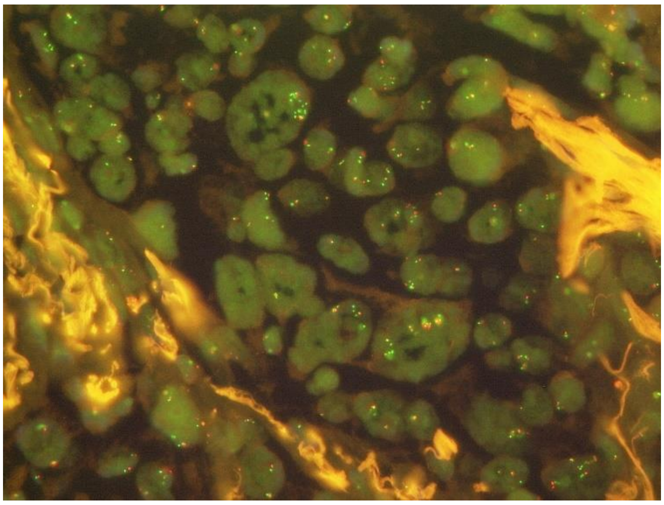

FGFR1 signals (60 cells)	Chr.8 signals (60 cells)	*FGFR1*-FISH-Ratio	*FGFR1*-FISH-copy-number (343/60)
343	177	1.9	5.7

Suppl. Figure 4:
Cloning of delta FGFR1

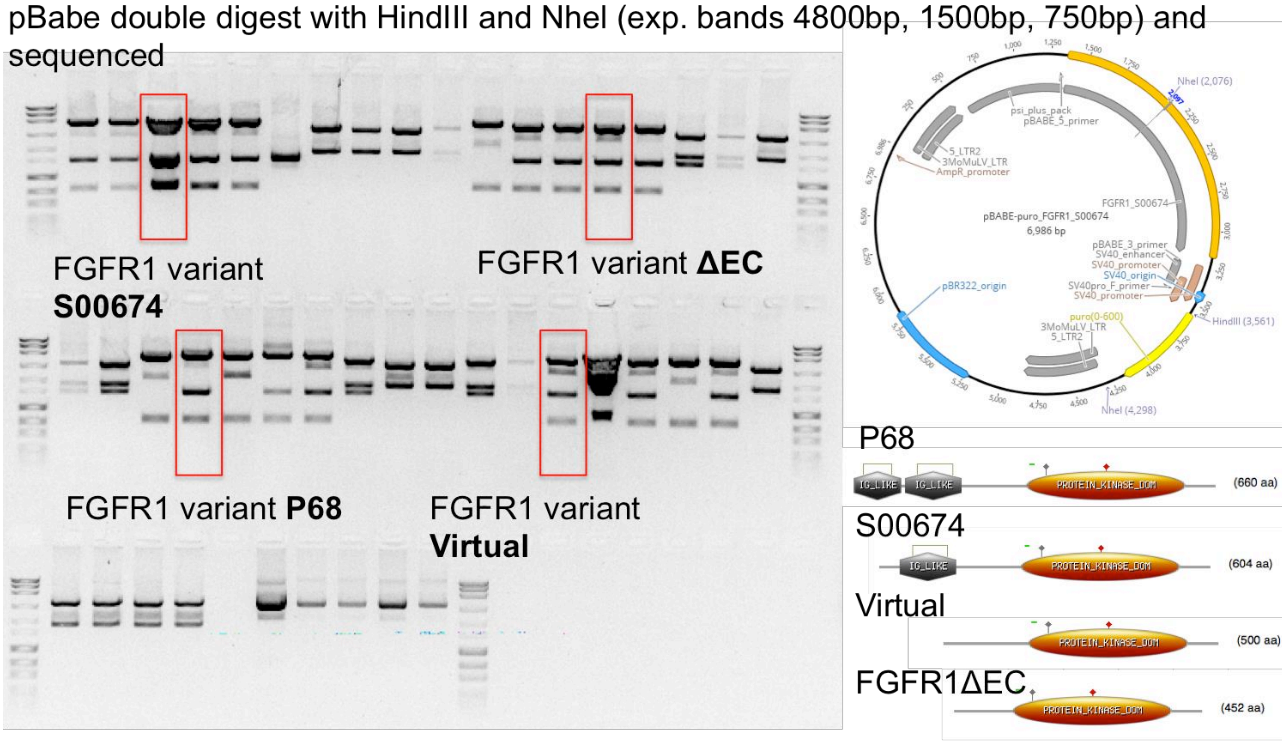

Suppl. Figure 5:
Oncogenic delta FGFR1 expression cause phosphorylated protein band shift

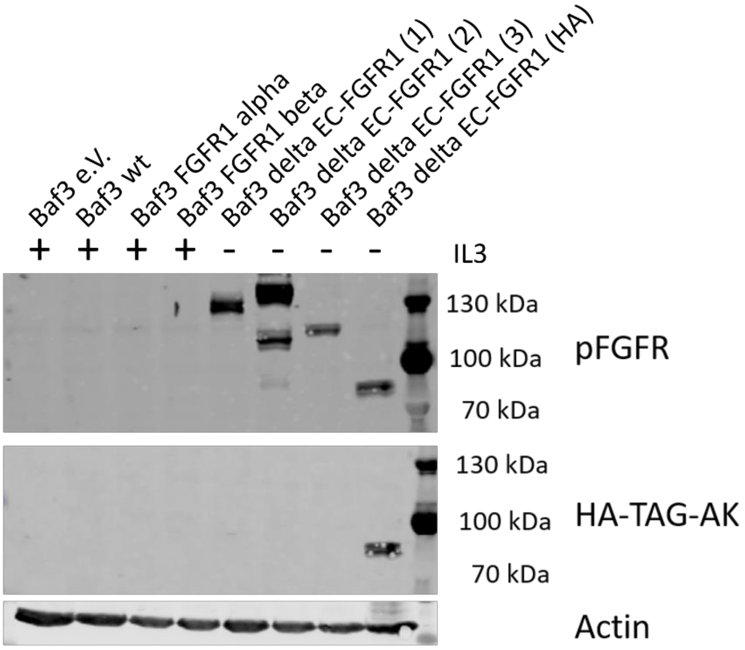

Suppl. Figure 6:
Baf3 cells RT-PCR Electropherogram

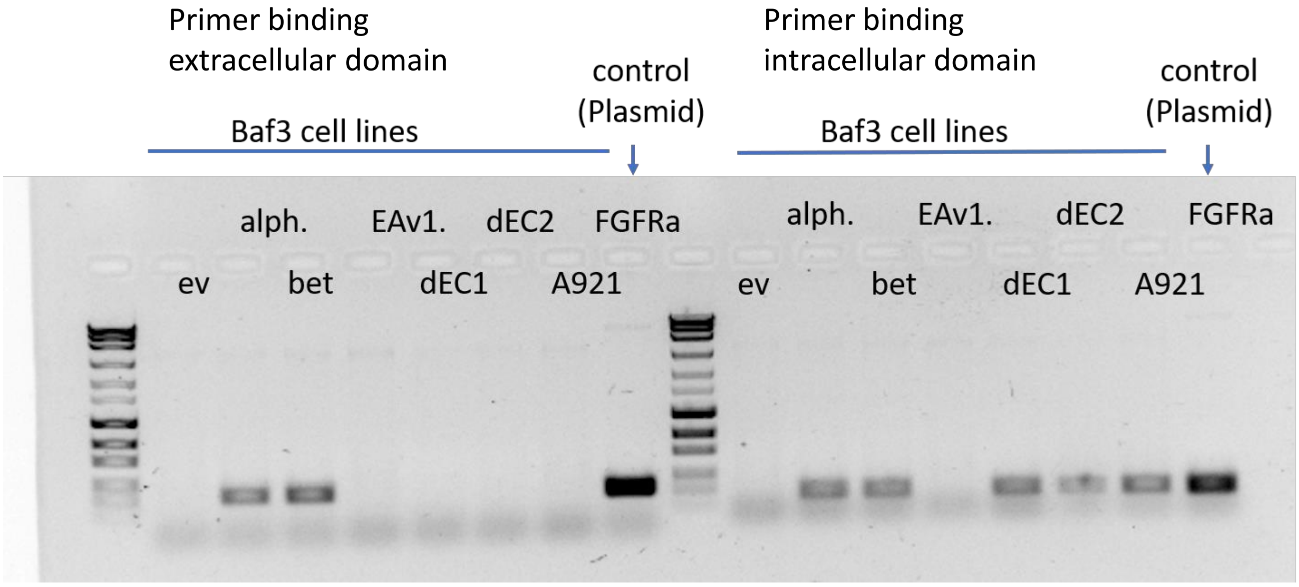

ev = empty vector
alph. = *FGFR1* alpha
bet = *FGFR1* beta
EVv1 = EML4-Alk version 1

dEC1 = delta EC *FGFR1* Experiment 1
dEC2 = delta EC *FGFR1* Experiment 2
A921 = delta EC FGFR1 Experiment 3
FGFR1a = FGFR1 alpha plasmid

Suppl. Figure 7:
Screening of Baf3 cells against Bromodomain inhibitors

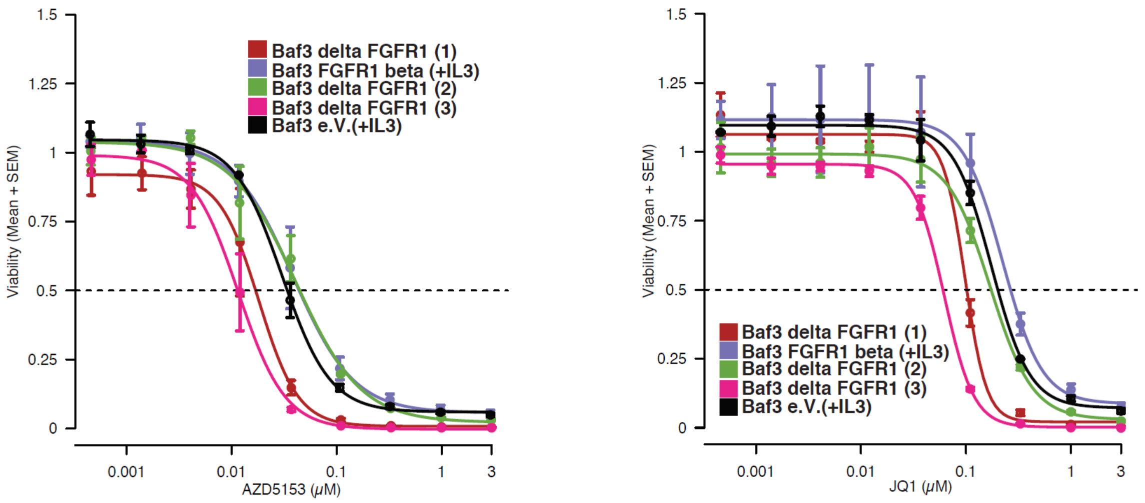

Suppl. Figure 8:
FGFR inhibitor sensitive cell lines

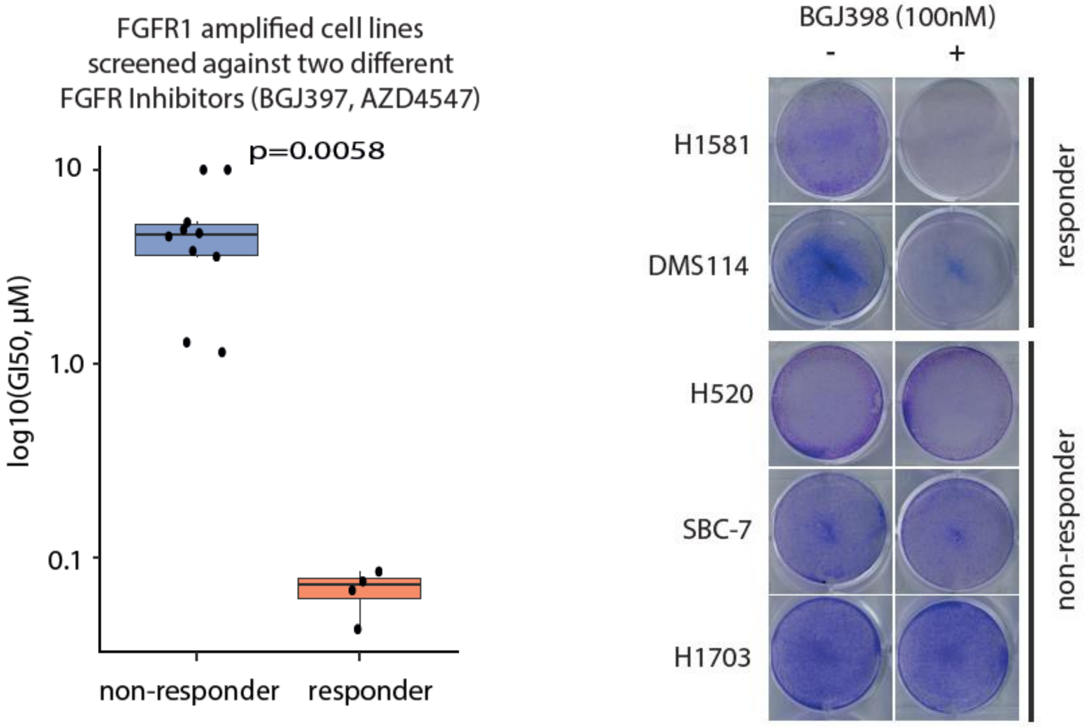

Suppl. Figure 9:
8p11-p12 amplification pattern correlates with FGFR inhibitor sensitivity

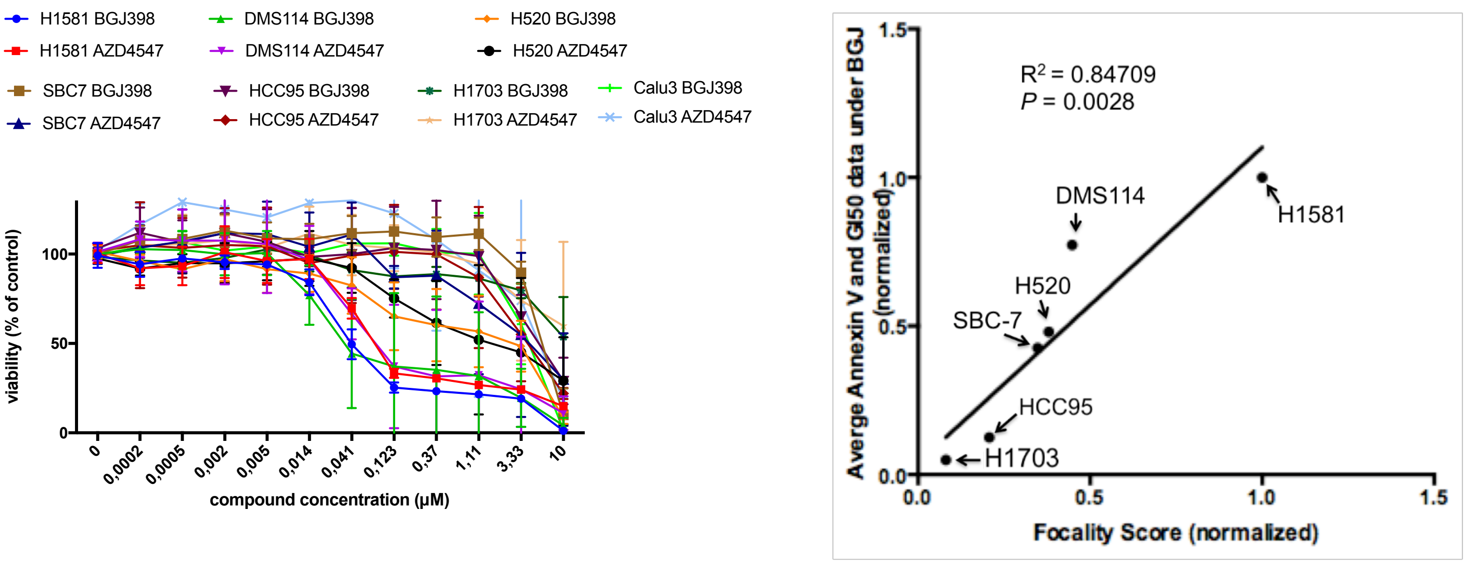

Suppl. Figure 10:
Individual copy number plot per gen and cell line

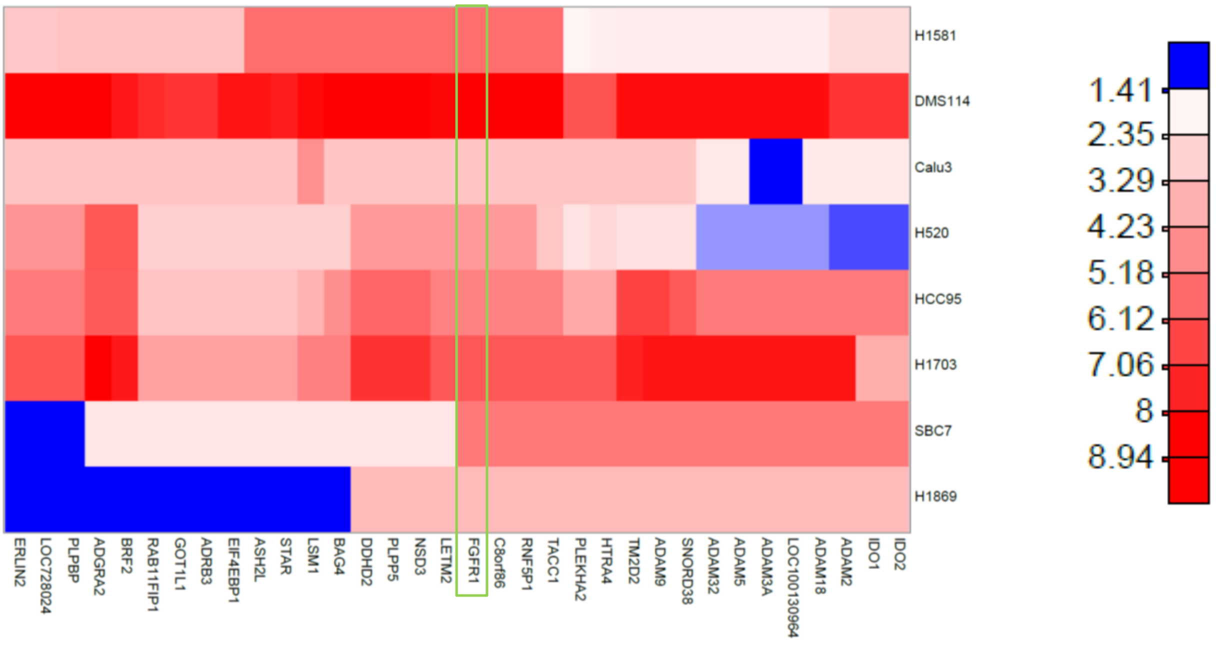

Suppl. Figure 11:
Individual copy number plot per gen and PDX

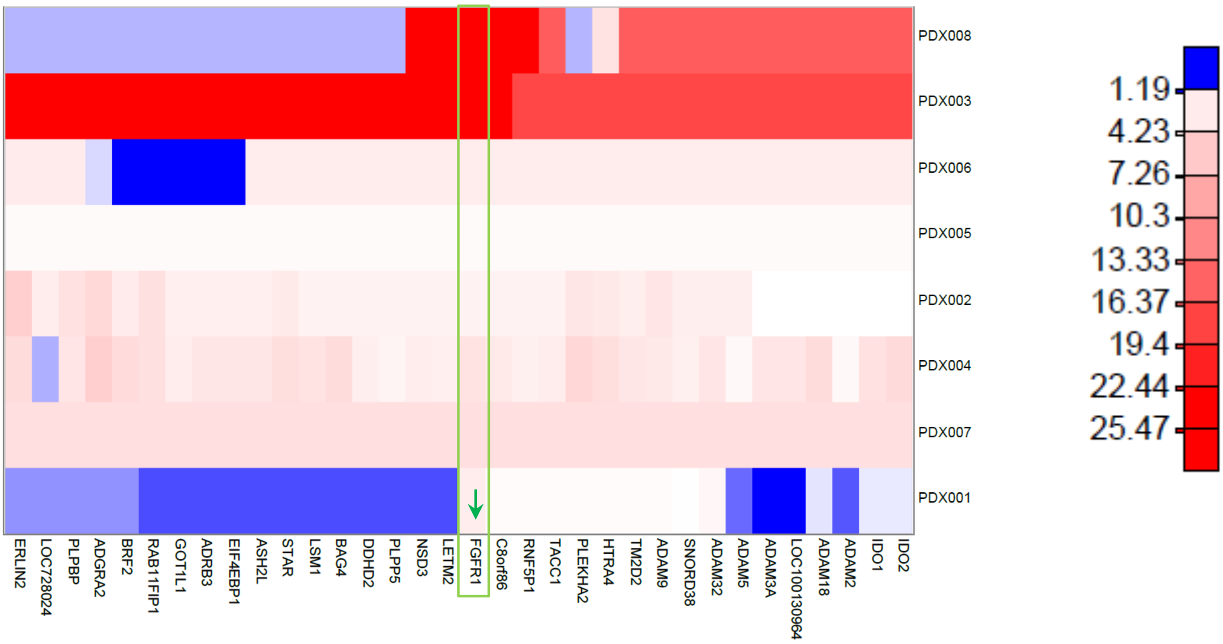

Suppl. Figure 12:
Inhibitor response of 8p-amplified patient derived xenografts

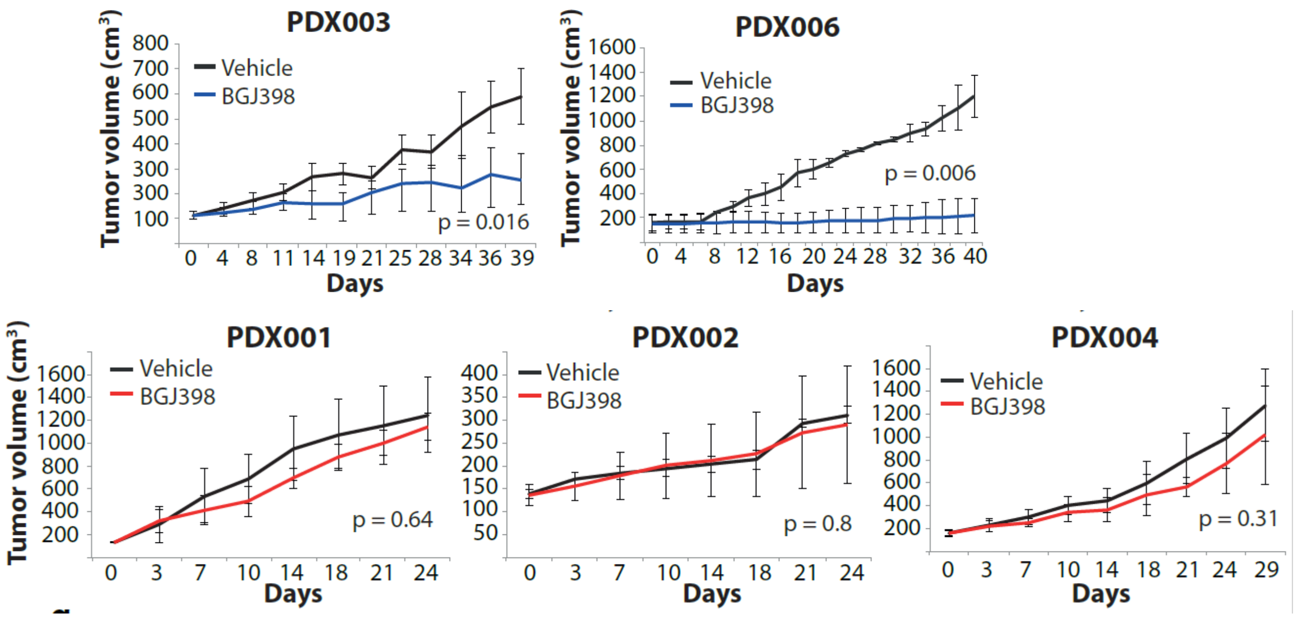

Suppl. Figure 13:
Copy number analysis of 8p11-p12 amplified SQLC samples

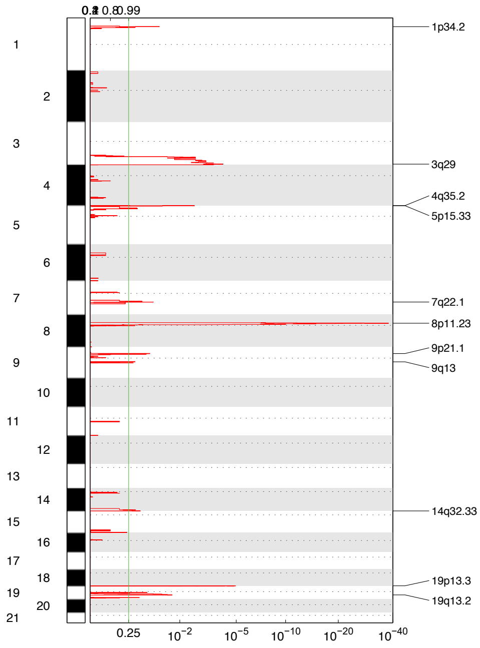

8p11.23: FGFR1, NSD3, LETM2, C8orf86
q = 1.3E-38

Suppl. Figure 14:
Amplification pattern of samples with a head-to-head break in NSD3

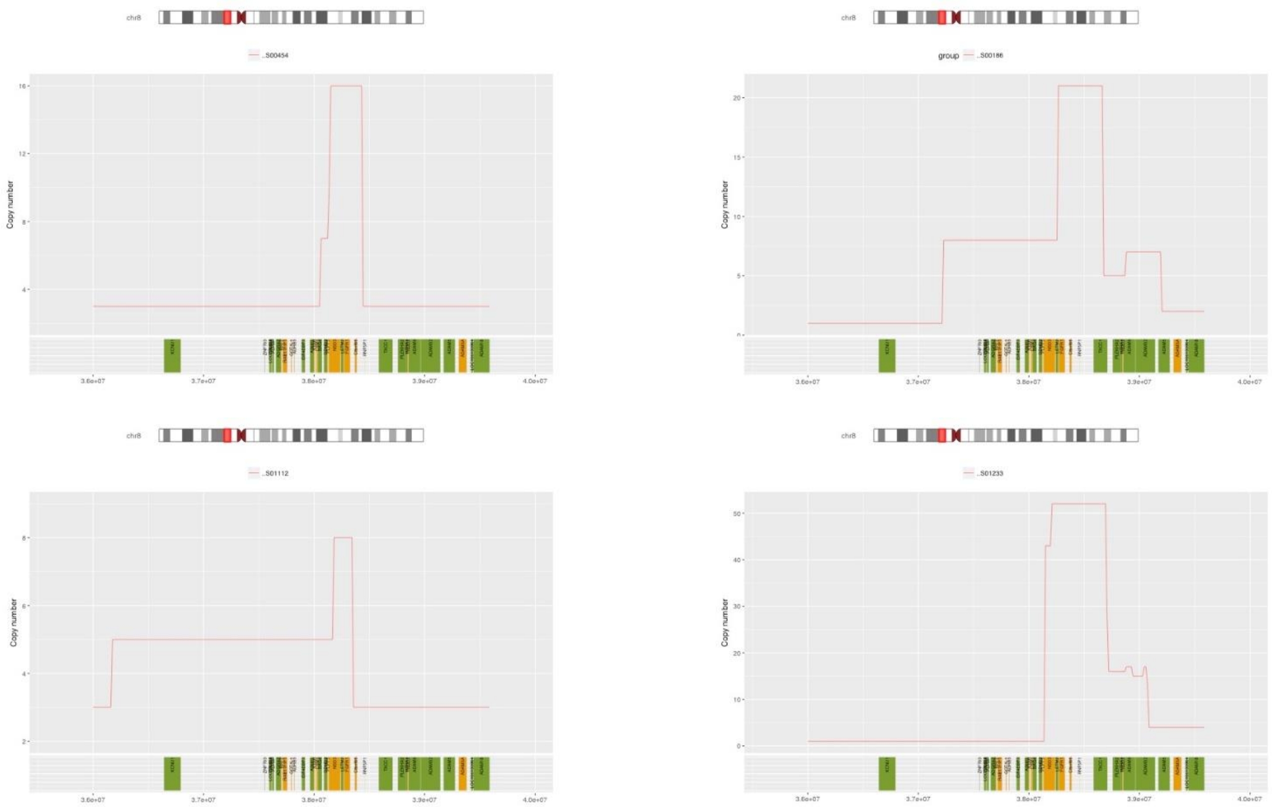

Suppl. Figure 15:
Transcription of *NSD3* long vs. short in samples with/without *NSD3* break

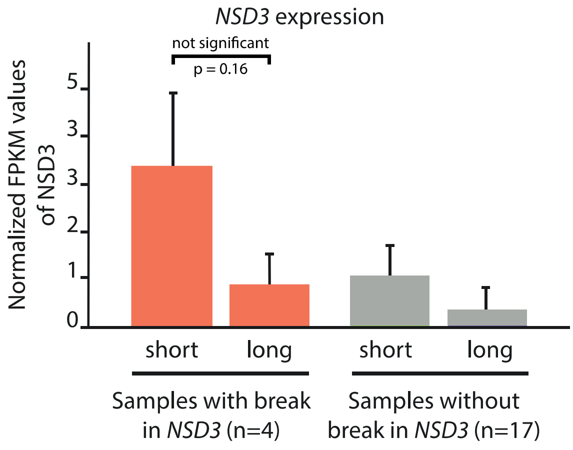

Suppl. Figure 16:
8p11-12 amplified lung cancer carcinomas show frequent telomeric losses

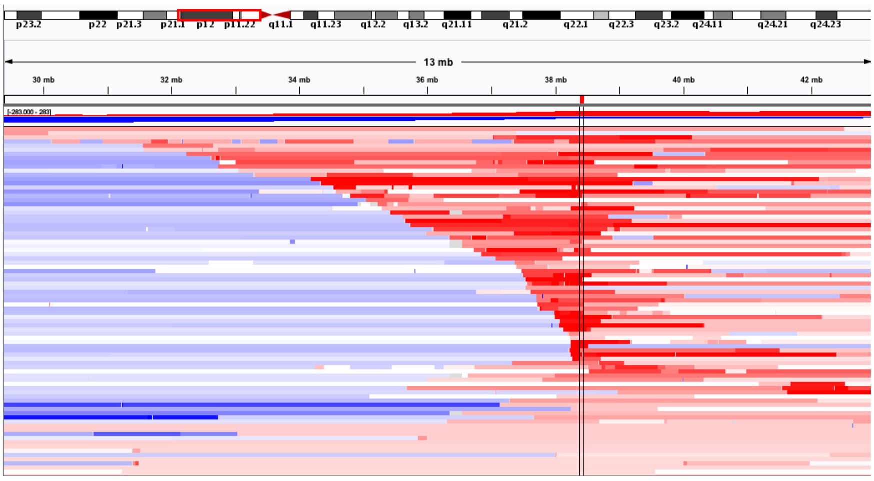

Suppl. Figure 17:
Copy number plot and detected breaks of on *FGFR1* amplified sample

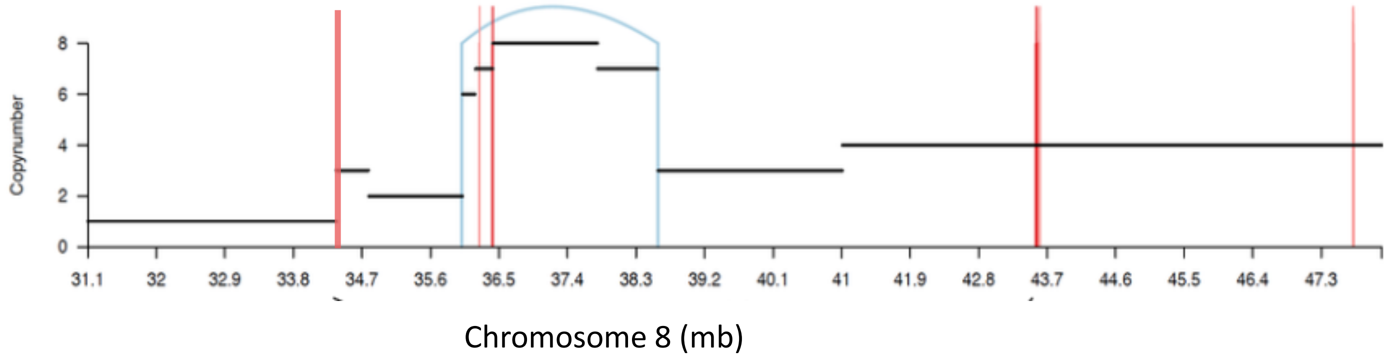

Suppl. Figure 18:
Copy number plot and detected breaks of on *FGFR1* amplified sample

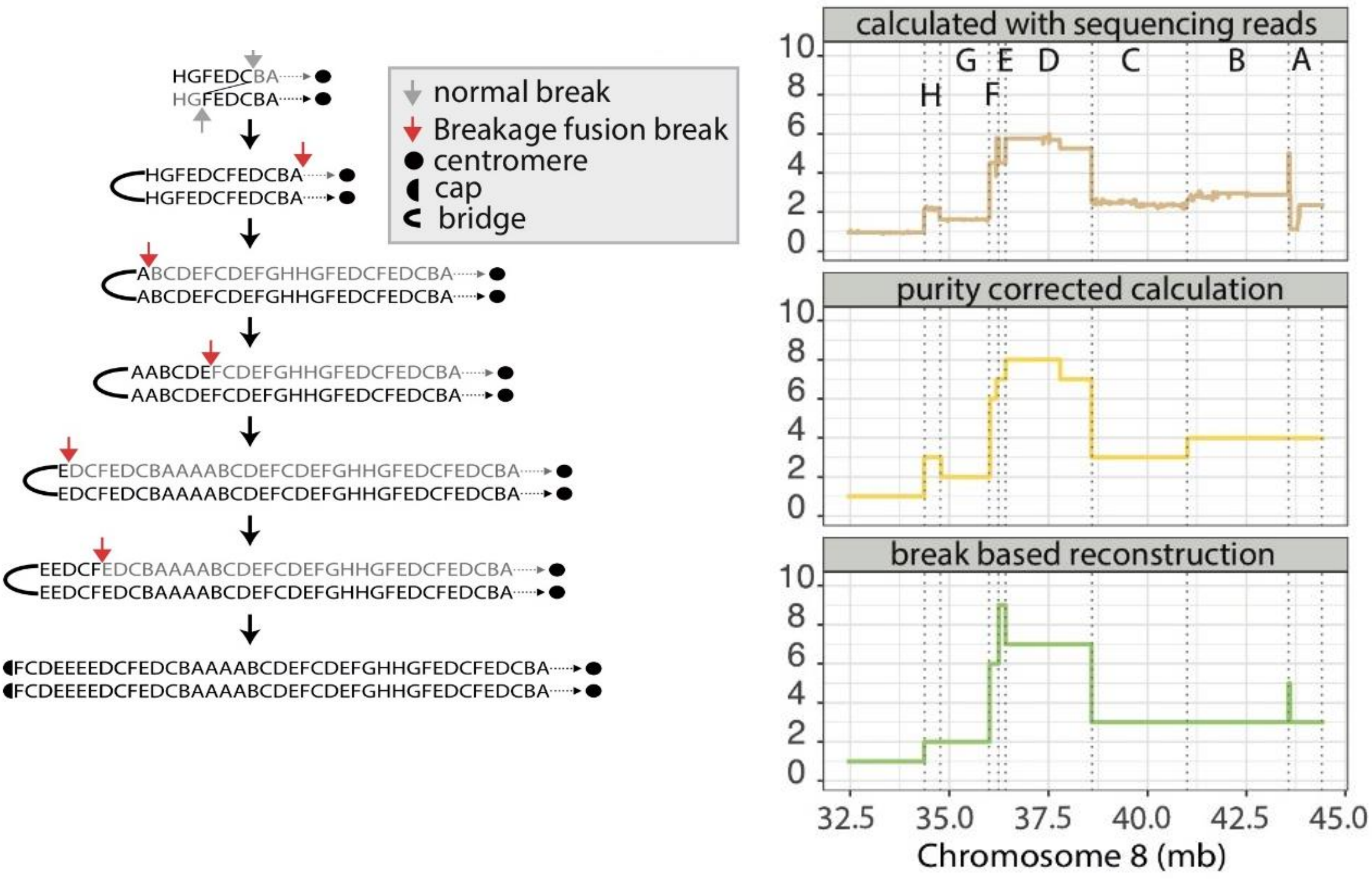

Suppl. Table 1:
Primer list

Name	Sequence
191_F1_S00674	CAGGGTGACTCGGCTCTCTA
203_R1_amplify_FGFR1	AAACAGACCAAACCGACAGG
193_F2_S00674	GGGAGGTAAAACTGGGATCG
204_R2_amplify_FGFR1	GCTGTAGCCCTGAGGACAAG
261_6_R1	CAGCAGATTTGATGCAGCTG
262_6_R2	CTGAGGTAGTTATTCGGAACACA
210_F_Virtuell_GA	agccctcactccttctctaggcgccggccgATGGAGGTGCTTCACTTAAGAAATG
211_R_FGFR1_GA	ctggcgaattcctacgtaccaccacactggTCAGCGGCGTTTGAGTCC
212_attB1_S00674	aaagcaggcttcCACCTGGAGCATCATAATGGA
213_attB1_S00674	aaagcaggcttcTGGAGCATCATAATGGACTCTG
214_attB1_A921	aaagcaggcttcCCGGCAGTGATGACCTCG
215_attB1_A921	aaagcaggcttcCAGTGATGACCTCGCCCC
216_attB1_p68	aaagcaggcttcTTGGACATCCCCAGAAAAGA
217_attB1_p68	aaagcaggcttcCCATATTGGACATCCCCAGA
218_attB1_FGFR1_Virtuell	aaagcaggcttcCCGACAAAGAGATGGAGGTG
219_attB1_FGFR1_Virtuell	aaagcaggcttcCAAAGAGATGGAGGTGCTTCAC
